# Stress-responsive and metabolic gene regulation are altered in low S-adenosylmethionine

**DOI:** 10.1371/journal.pgen.1007812

**Published:** 2018-11-28

**Authors:** Wei Ding, Daniel P. Higgins, Dilip K. Yadav, Adwait A. Godbole, Read Pukkila-Worley, Amy K. Walker

**Affiliations:** 1 Program in Molecular Medicine, UMASS Medical School, Worcester, MA, United States of America; 2 Department of Computer Sciences, Georgia Institute of Technology, Atlanta, GA, United States of America; 3 Program in Innate Immunity, Division of Infectious Diseases and Immunology, UMASS Medical School, Worcester, MA, United States of America; Stanford University, UNITED STATES

## Abstract

S-adenosylmethionine (SAM) is a donor which provides the methyl groups for histone or nucleic acid modification and phosphatidylcholine production. SAM is hypothesized to link metabolism and chromatin modification, however, its role in acute gene regulation is poorly understood. We recently found that *Caenorhabditis elegans* with reduced SAM had deficiencies in H3K4 trimethylation (H3K4me3) at pathogen-response genes, decreasing their expression and limiting pathogen resistance. We hypothesized that SAM may be generally required for stress-responsive transcription. Here, using genetic assays, we show that transcriptional responses to bacterial or xenotoxic stress fail in *C*. *elegans* with low SAM, but that expression of heat shock genes are unaffected. We also found that two H3K4 methyltransferases, *set-2/SET1* and *set-16/MLL*, had differential responses to survival during stress. *set-2*/SET1 is specifically required in bacterial responses, whereas *set-16/MLL* is universally required. These results define a role for SAM in the acute stress-responsive gene expression. Finally, we find that modification of metabolic gene expression correlates with enhanced survival during stress.

## Author summary

Animals respond to stress by activating suites of protective genes. A specific metabolite, S-adenosylmethionine (SAM), influences how these genes are activated in a variety of stress conditions. SAM is produced by the 1-carbon cycle and is the major donor for methylation reactions. Thus, SAM is used in the modification of histones, DNA, RNA and production of phospholipids. Here, we show that *C*. *elegans* with low SAM have reduced responses to a bacterial and toxic stress, but respond normally to heat stress. We also analyzed how animals that have reduced activity in some of the enzymes that use SAM to modify histones might respond to stress. One enzyme, SET-2, was needed only for survival in bacterial stress, whereas the other related enzyme, SET-16, was universally required. The availability of SAM may be affected by diets low in choline or methionine, alcohol or diseases such as cystic fibrosis. Thus, low SAM availability may leave organisms less able to respond to additional stress, which could exacerbate tissue injury or disease progression.

## Introduction

Cellular functions are profoundly affected by metabolic state. For example, transcriptional regulation can be linked to metabolism through the modification of chromatin by methylation [1]. Using the methyl groups produced by the 1-carbon cycle (1CC) and donated by S-adenosylmethionine (SAM), histone methyltransferases (HMTs) can change the regulatory state of chromatin, promoting or limiting gene activity [2]. HMT activity can be controlled by recruitment of HMT containing complexes to specific genomic locations [2]. However, SAM availability may also affect histone methylation patterns [3]. SAM is produced by the 1-carbon cycle (1CC) and levels can be affected by folate, methionine or choline levels or by other factors such as alcohol consumption [4]. Variations in SAM levels have been proposed to mediate transgenerational inheritance of epigenetic patterns or other gene regulatory events, however, a direct mechanistic connection has been difficult to establish [5]. Although SAM is necessary for all histone methylation events, *in vivo* studies have suggested that particular methylation marks are more sensitive to changes in SAM levels. For example, induced pluripotent stem cells (iPSCs), murine liver, and *C*. *elegans* all show a decrease in H3K4me3 levels as SAM levels drop [6–9]. Furthermore, in budding yeast, a rise in SAM levels is followed by increases in H3K4me3 [10]. Furthermore, these linked changes in SAM levels and H3K4me3 also correlate with changes in cell-type-specific gene expression and differentiation in iPS cells [7]. Finally, Dai et al. have recently demonstrated that treatments with high and low methionine, which is the precursor for SAM, alters H3K4me3 peak width at genes in steady-state conditions in mouse liver and human cancer cells [11]. Thus, SAM levels are tightly linked to H3K4me3 dynamics.

Trimethylation of H3K4 is a common modification occurring close to the start site of actively transcribed genes and is accomplished through the activity of the COMPASS complex [12]. The KTM2 family of HMTs serves as the enzymatic activity of COMPASS providing mono, di and trimethylated states [13]. In yeast, there is a single member, Set1, whereas mammals can use one of seven enzymes, within subfamilies of SET1, MLL (Mixed lineage leukemia) or THX (Trithorax) [12]. However, the relationship between H3K4me3 and transcription is complex, as it does not appear to be necessary for global gene expression in basal conditions [14]. In yeast, Set1 has an important role in limiting the expression of ribosomal genes during the response to diamide stress [15] suggesting that chromatin-modifying factors are especially critical when organisms experience stress. The H3K4 methyltransferase family in mammals appears to have overlapping as well as specialized functions in either specificity for mono, di or tri-methylation or through distinct roles in development [13]. However, clearly defined roles for each MT have been difficult to discern.

*C*. *elegans* genome encodes a simplified KTM2 family containing three H3K4 methyltransferases, *set-2*/SET1, *set-16*/MLL and *ash-2*/THX [16]. Interestingly, these methyltransferases have distinct developmental and tissue-specific biological functions. *set-2*/SET1 is broadly important for H3K4 trimethylation in embryos and the germline [17, 18] and the intestine [8]. Also, loss or reduction of *set-2/*SET1 influences fertility across generations [19], lifespan [20] and lipid accumulation [21]. *ash-2* acts through the germline to affect lifespan and lipid accumulation in the intestine [20–22]. *set-16*/MLL, on the other hand, appears to be dispensable for H3K4me3 in the early embryo and germline (Li, 2011), while we found that it has a partial requirement in the adult intestine [8]. Thus, while H3K4me3 marks the start sites of actively transcribed genes, the methyltransferases producing it can have diverse and long-acting biological effects.

Using a *C*. *elegans* model of low SAM, we previously found that transcriptional responses to a bacterial pathogen failed and these bacterial-response genes did not show the normal pattern of H3K4me3 close to the transcriptional start sites, [8]. We also found that the HMT *set-16*/MLL was required for full induction, whereas *set-2*/SET1 appeared dispensable [8]. We hypothesized that animals with low SAM might fail to transcriptionally respond to stress and that the HMTs may also have distinct roles in modulating stress responses. In our present study, we set out to compare induction of transcriptional responses and survival upon stress exposure between *C*. *elegans* with reduced SAM and animals with limited H3K4me3 function. Because distinct stresses may rely on different transcriptional activation mechanisms, we also compared whole-genome expression patterns in three stresses: pathogenic bacteria, xenotoxic and heat. We found that the induction of genes in the pathogen and xenotoxic stress response were diminished in low SAM, with concomitant reductions in survival in these animals. However, while pathogen and xenotoxic-stress genes were affected after both *set-2*/SET1 and *set-16/*MLL RNAi, *set-16*/MLL was uniquely required for survival in all three stresses. This suggests SAM and *set-16* have essential functions in transcriptional responses to diverse stresses. Interestingly, induction of heat stress response genes, which are controlled primarily by promoter pausing of RNA Pol II [23], occurs even in low SAM and after H3K4 methytransferase knockdown. While expression of canonical heat shock genes occurred in each of these conditions, *sams-1* animals fared better than controls, *set-2* animals survived at control levels and *set-16* animals died rapidly. Thus, the expression of stress response genes and survival may not correlate in all cases. Finally, we find that in addition to stress-responsive genes, regulation of metabolic genes may be key to the survival of animals with deficient H3K4 methylation during stress.

## Results

### Large-scale changes in stress-induced gene expression in low SAM

**Fig 1:**
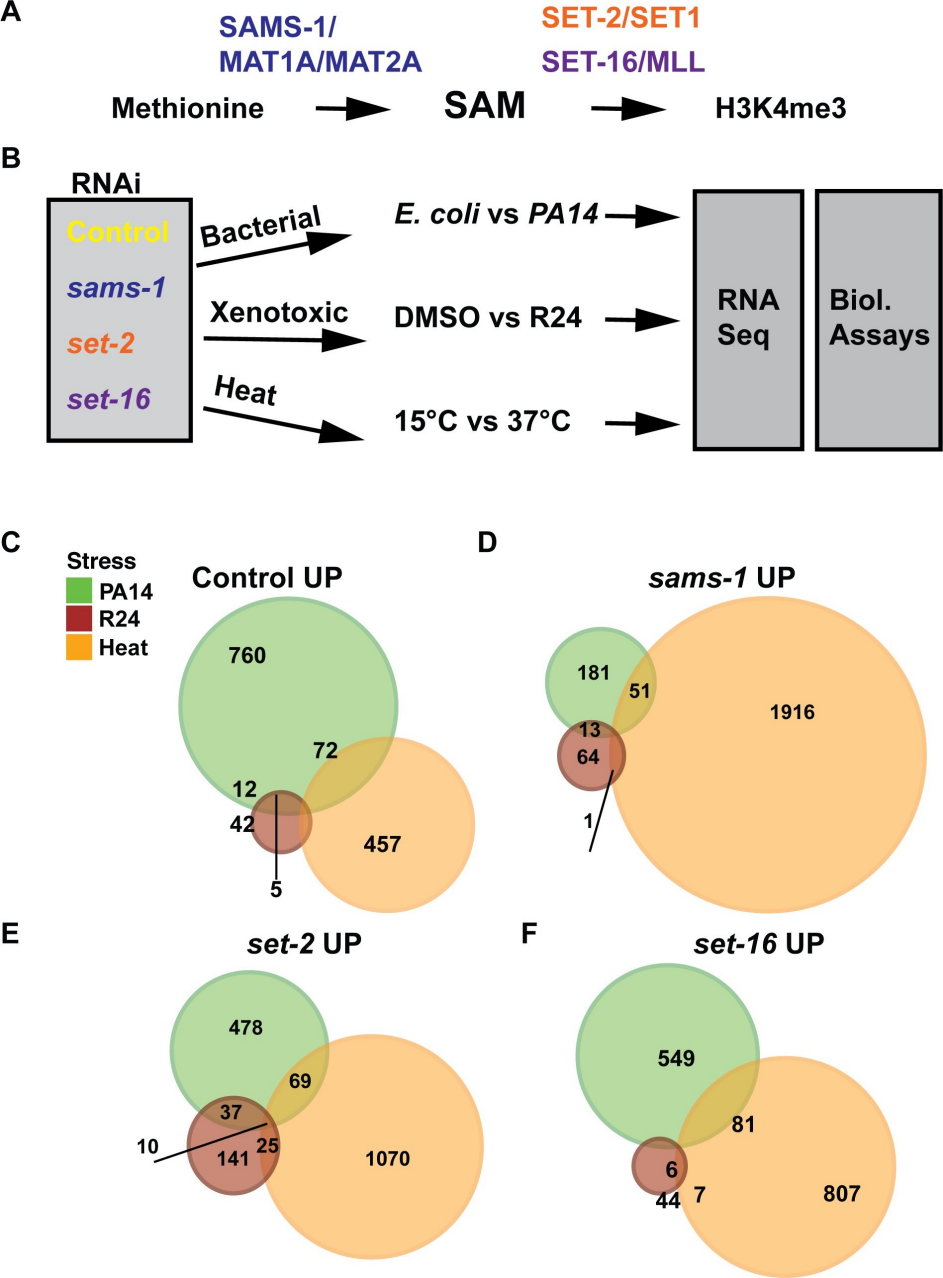
SAM plays an important role in the transcriptional response to stress. **A.** Schematic showing the metabolic link between s-adenosylmethionine (SAM) production and histone methylation **B.** Diagram of the experimental rationale comparing knockdowns of the SAM synthase *sams-1* and the H3K4 methyltransferases *set-2*/SET1 and *set-16*/MLL in the transcriptional response to three distinct stresses. Venn diagrams comparing stress responsive gene expression in control (**C**), *sams-1* (**D**) *set-2* (**E**) and *set-16* RNAi upregulated genes (**F**). Upregulated genes were defined as increased by 2 or more fold with an FDR of less than 0.01 in each of the stresses.

Gene regulatory events can be controlled by histone methylation; however, it is not clear how levels of the methyl donor SAM may alter methylation patterns and gene expression in different physiological conditions (**Fig 1A**). In *C*. *elegans*, we previously found animals with a mutation in the SAM synthase *sams-1*, which have 50% of the SAM of wild-type animals [24], had poor survival on the bacterial pathogen *Pseudomonas aeruginosa* [8]. SAM deficient animals failed to upregulate selected pathogen-response genes and had reduced global H3K4 trimethylation in intestinal nuclei as well as at specific pathogen-response genes [8]. We hypothesized this could represent a general failure of stress-responsive gene expression, as low SAM levels were unable to support rapid remodeling of H3K4 methylation as transcriptional needs changed. To test this model (**Fig 1B**), we used RNAi to knockdown *sams-1* or the H4K4me3 methyltransferases (HMTs) that use SAM, *set-2* and *set-16*, then exposed animals to three stresses: bacterial (*P*. *aeruginosa)* xenotoxic, or heat. For xenotoxic stress, we used R24, an agent that robustly stimulates both immune and detoxification responses in *C*. *elegans* [25–27]. Next, we used whole genome RNA sequencing to determine which genes changed in each stress and assayed how they were affected by low SAM or depletion of the HMTs and selected genes with greater than two-fold change in any of the conditions with a false discovery rate (FDR) of <0.01 for further analysis. To determine if gene expression patterns were shared between control, *sams-1*, *set-2* and *set-16* animals in response to *P*. *aeruginosa*, we mapped gene expression patterns with Venn diagrams for up (**Fig 1C–1F**) and down (**S1A–S1D Fig**) regulated genes. We found distinct, large scale gene expression changes with each stress, suggesting gene expression modules were specified by stress-specific mechanisms rather than by SAM or these H3K4 methyltransferases.

### Role of SAM in gene expression and H3K4 methylation in basal conditions

We first examined gene expression changes between control and *sams-1(RNAi)* in non-stressed conditions (basal). SAM may contribute to PC production as well as to histone methylation (**S2A Fig**) and several lines of evidence from our previous study of gene allow us to distinguish indirect effects downstream of phospholipid methylation from other SAM-dependent events. In basal conditions, several hundred genes changed by more than two-fold after *sams-1(RNAi)* (**S2B Fig, S1 Table**), with significant overlap with our previous microarray results [8]. In that study, we found that most gene expression changes were linked to methylation-dependent PC production, as they were returned to wild-type levels when PC levels were rescued by dietary choline (**S2C Fig**) [8]. Stress-responsive genes activated downstream of PC were (1) expressed at low levels (2–5 fold) and (2) expression was returned to wild-type levels by dietary choline, which rescued PC levels [8]. This is in contrast to activation of bacterial stress-responsive genes by *P*. *aeruginosa*, which was dynamic (up to 250 fold) and not responsive to choline [8]. Our observation that *P*. *aeruginosa*-responsive gene expression also depended on H3K4 methyltransferases suggests that stress responsive transcription might have a distinct requirement for SAM and H3K4 methyltransferases than in basal conditions. Finally, decreases in H3K4me3 in *C*. *elegans* intestinal nuclei were not rescued when choline returned PC to wild type levels, suggesting that SAM-dependent decreases in this histone methylation mark are not linked to indirect effects from PC.

Both H3K4 methylation and PC production are significant consumers of SAM [3] (**S2A Fig**). Recently Ye, et al. show that H3K4 tri-methylation can increase in *Saccharomyces cerevisiae* when PC production is blocked and SAM levels increase [10]. In agreement with these findings, we also observed that global H3K4me3 levels increase when the PC-producing methyltransferases *pmt-2* was knocked down (**S2D Fig**). Thus, in basal conditions, gene expression changes to compensate for decreases in PC are the predominant effect of *sams-1* loss, with negligible effects due to other methylation pathways. Finally, modified H3K4 may exist in several methylation states [13]. Using immunostaining with antibodies to H3K4me1 and H3K4me2, we found that levels did not decrease as they had with H3K4me3 (**S2E Fig**; [8]), suggesting that the trimethylated state is most sensitive to SAM levels in adult *C*. *elegans* intestine.

### SAM is important for the transcriptional response to a bacterial stress

**Fig 2:**
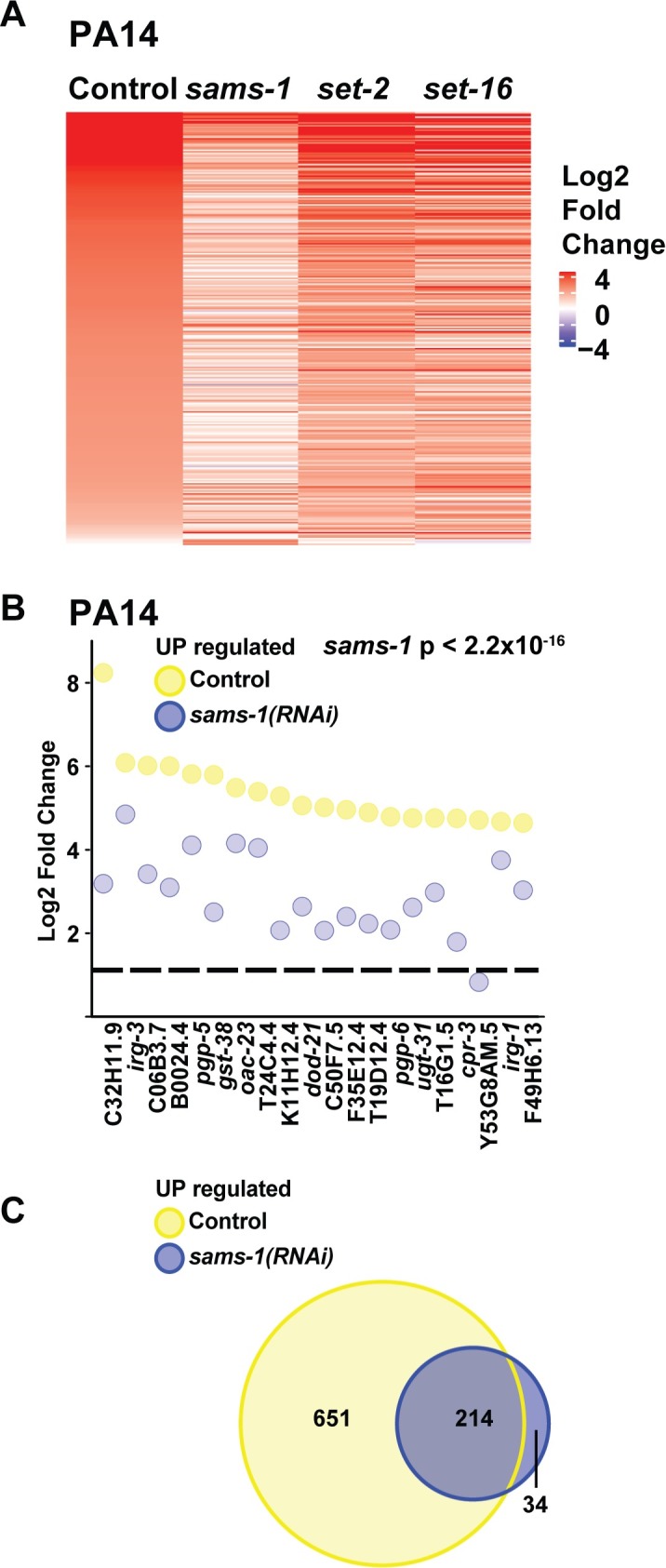
Transcriptional response to *P*. *aeruginosa* requires *sams-1*. (**A**) Heat map showing genes upregulated by more than 2-fold with an FDR of less than 0.01 in *C*. *elegans* exposed to *P*. *aeruginosa*. (**B**) Strip-plot comparing the top 20 genes upregulated in control vs. *sams-1(RNAi)* animals exposed to *P*. *aeruginosa*. The dotted line is placed at one on the Y-axis. Statistical significance calculated by KS value. (**C**) Venn Diagram comparing the overlap between genes upregulated more than 2-fold in control vs. *sams-1(RNAi)* animals exposed to *P*. *aeruginosa*.

Next, we compared gene expression patterns in control, *sams-1*, *set-2* and *set-16* RNAi animals during *P*. *aeruginosa* exposure. Control animals upregulated 651 genes more than two-fold in response to the bacterial stress (**Fig 2A and 2B, S2 Table**) with a high concordance to previous studies that identified *P*. *aeruginosa*-response genes [28, 29] (**S2 Table**). Heat maps comparing genes upregulated more than 2-fold with an FDR of < 0.01 show lower induction after *sams-1* RNAi, with intermediate effects after *set-2* or *set-16* knockdown (**Fig 2A**). Focusing on the top 20 expressed genes in control animals, we find a significant reduction in expression (**Fig 2B**) and finally, we find that few genes outside the pathogen response are induced after *sams-1* RNAi (**Fig 2C**). The transcriptional response to *P*. *aeruginosa* also includes downregulation of a small subset of genes [28]. Comparisons between control and *sams-1(RNAi)* gene expression patterns show that a proportion of the top 20 downregulated genes in control animals fail to decrease after *sams-1* RNAi (**S3A Fig**) and that only about 5 percent of these genes overlap (**S3B Fig**). Thus, this whole genome data confirms our analysis of selected *P*. *aeruginosa*-responsive genes [8] and shows that SAM is essential for the broad transcriptional changes occurring during stress caused by a pathogenic bacteria, reducing both total numbers of regulated genes and their magnitude.

### Transcriptional responses and survival upon xenobiotic challenge are diminished in low SAM

**Fig 3:**
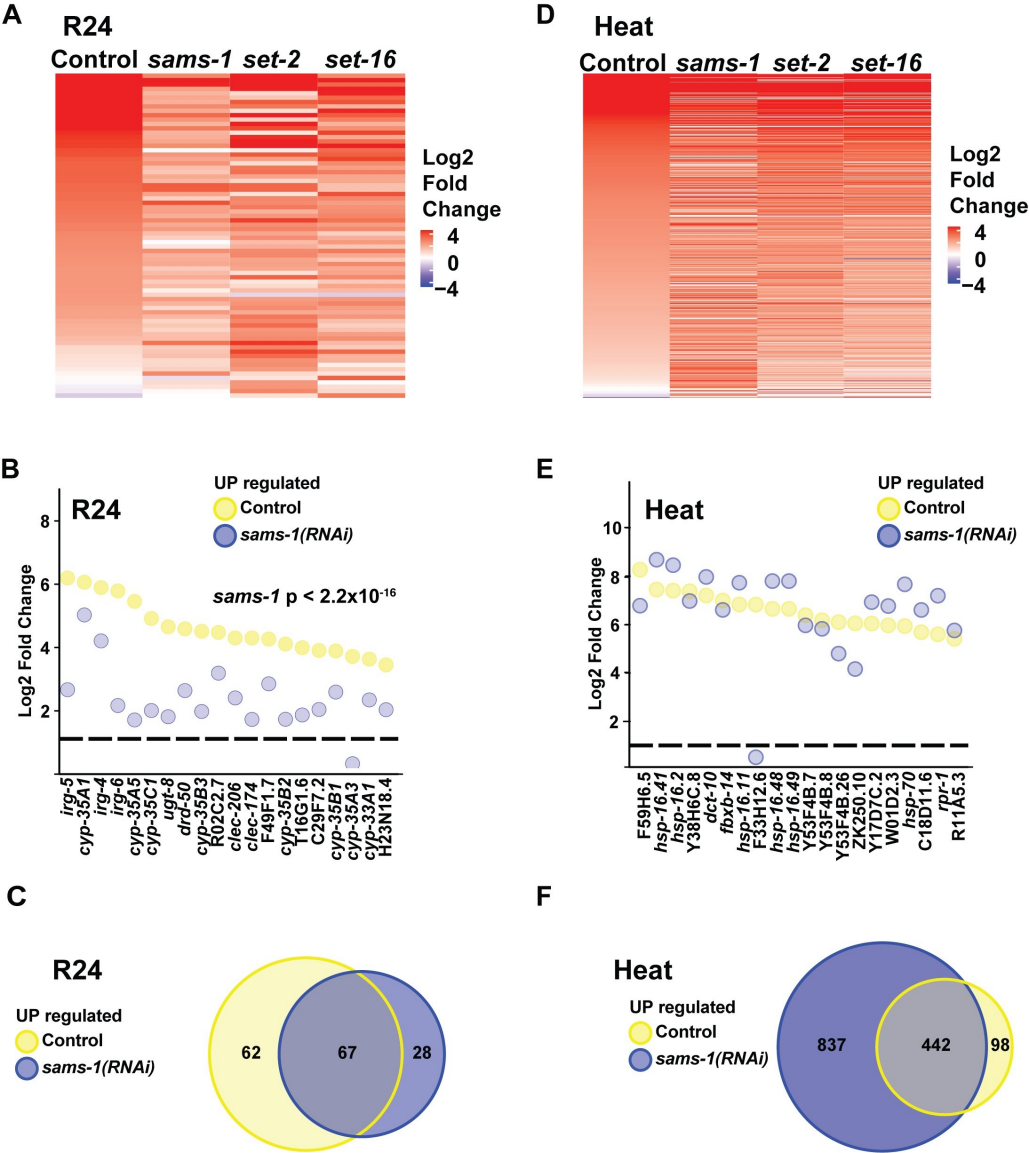
Differential transcriptional responses to a xenotoxic stress and heat stress after *sams-1(RNAi)*. (**A**) Heat map showing genes upregulated by more than 2-fold with an FDR of less than 0.01 in *C*. *elegans* exposed to R24. **(B)** Strip plots showing that the top 20 genes upregulated in controls in response to R24 decreased are in *sams-1(RNAi)* animals. (**C**) Venn diagrams show that the majority of genes upregulated more than two-fold in *sams-1* animals in response to R24 are also upregulated in controls. (**D**) Heat map showing genes upregulated by more than 2-fold with an FDR of less than 0.01 in *C*. *elegans* exposed to heat. (**E**) Strip-plot shows that the top 20 genes expressed after heat shock are upregulated similarly in control and *sams-1(RNAi)* animals. (**F**) Venn Diagram comparing gene sets regulated after heat stress shows many ectopic genes upregulated in *sams-1(RNAi)* animals.

To determine how *sams-1* RNAi animals respond to a distinct stress, we treated control and *sams-1* RNAi animals with R24, a xenotoxic agent that induces both detoxification and innate immune defenses [25–27]. R24 was originally identified in a screen of 37,200 small molecules that utilized *C*. *elegans* as a heterologous host to identify new anti-infective compounds[30]. Interestingly, R24 protects nematodes from bacterial infection by boosting the transcription of innate immune defenses [26, 27]. This molecule is also toxic to worms growing under normal laboratory conditions. Exposure to R24 strongly activates the transcription of cytochrome P450 and other detoxification genes, it shortens nematode lifespan and delays worm development [26, 27]. We used R24 as a tool to compare gene expression changes in animals with low amounts of SAM. RNA-seq was performed on control and *sams-1(RNAi)* after treatment with R24. A set of genes was significantly upregulated by R24 in wild-type animals, which was consistent with published results [25–27]. Importantly, the induction of genes by R24 was significantly attenuated in *sams-1(RNAi)* animals (**Fig 3A, S3 Table**). The 20 most highly induced genes after R24 treatment included multiple cytochrome p450s as well as previously identified pathogen response genes (**Fig 3B**) [27]. Strip plots comparing levels in control and *sams-1(RNAi)* animals show that each gene was markedly decreased (**Fig 3B**). We also found that most of the genes induced in *sams-1* animals were part of the response to R24 in control animals (**Fig 3C**). R24 also induces the downregulation of a limited subset of genes [27]. Comparison between genes downregulated in control animal or after *sams-1* RNAi shows that *sams-1* RNAi also limits this downregulation (**S3C and S3D Fig**). Thus, low SAM attenuates the transcriptional response to a xenotoxic agent, just as it does to bacterial stress-responsive gene expression induced by *P*. *aeruginosa*.

### Heat shock transcriptional response occurs independently of SAM

Transcriptional response to bacteria or xenotoxic agents are predicted to follow a classic signal transduction pathway where the extracellular stimulus activates a cellular signaling pathway linked to individual transcription factors and upregulation of stress-specific gene expression [31]. However, other stress-responsive genes expression, such as the heat shock genes, are regulated differently. RNA Pol II is paused at promoters of many heat shock genes and released into its elongating form in response to heat [32]. To determine if low SAM had the same effects on heat shock-dependent transcription as the bacterial or xenotoxic stress, we performed whole genome RNA sequencing on control, *sams-1*, *set-2* and *set-16* RNAi animals exposed to 37°C for one hour. We found that heat-shock genes such as *hsp-16*.*41*, *hsp-16*.*2*, *hsp-16-11*, *hsp-16*.*48*, *hsp-16*.*49* and *hsp-70* were strongly induced in control animals in these conditions (**Fig 3D–3F, S4 Table**).

In contrast to the bacterial or xenotoxic stress responses, comparison of control and *sams-1* patterns for genes induced at least 2-fold shows similar patterns (**Fig 3D**), suggesting that reduced SAM availability does not compromise the activation of heat-stress induced genes. Strip plots comparing expression of the top 20 genes activated in control animals compared to *sams-1* shows that most of the highly expressed genes are similarly or more highly expressed after *sams-1* RNAi (**Fig 3E**). Finally, Venn diagrams confirm that the genes upregulated after heat shock in controls are also upregulated in *sams-1* animals and that many genes ectopic to the heat shock response also increase (**Fig 3F**), suggesting that additional gene expression are activated in *sams-1* animals under heat stress. Control animals downregulated approximately 300 genes after heat shock; strikingly, nearly 2000 genes decreased in parallel *sams-1* RNAi animals (**S3E and S3F Fig**). Thus, while expression of heat shock genes seems to occur independently of SAM, other genes outside this classical response dramatically increase or decrease during heat shock in low SAM.

### Differential attenuation of stress-responses after knockdown of the H3K4 methyltransferases *set-2*

Histone methyltransferases use SAM to modify specific histone residues, modifying the chromatin environment to provide distinct gene regulatory states. Yeast contain a single H3K4 HMT, which provides mono, di and trimethylated states [12] and functions within the COMPASS HMT complex. Mammals encode 7 H3K4 HMTs that have different specificity for methylation states [12]. However, the non-redundant biological functions have been difficult to discern. *C*. *elegans* contains 3 H3K4 HMTs that affect H4K4me3, *set-2*/SET1, *set-16*/MLL and *ash-2*/THX. These HMTs affect embryonic and germline development [17, 18] and transgenerational inheritance through the germline [20, 22]. In our previous studies, we investigated the roles of *set-2*/SET1 and *set-16*/MLL in the adult *C*. *elegans* intestine, which is a critical tissue in the pathogen response [33]. Because H3K4me3 has been associated with dynamically transcribed genes and our previous results showing an increase in H3K4 tri-methylation at promoters of selected *P*. *aeruginosa* responsive genes during infection [8], we sought to determine if *set-2*/SET1 or *set-16*/MLL were downstream of SAM-dependent responses during the stress response. Neither *set-2* or *set-16* RNAi significantly affected gene expression in non-stressed conditions (**S4A and S4B Fig, S1 Table**). In parallel with *sams-1(RNAi)* RNA experiments (**Figs 1 and 2**), we exposed *set-2(RNAi)* animals to *P*. *aeruginosa*, the xenotoxic agent R24 or heat stress, extracted RNA and performed RNA-sequencing. Unbiased hierarchical clustering analysis of all genes significantly upregulated by any of these stresses showed that *set-2* and *set-16* RNAi grouped within each stress, suggesting similar overall gene expression patterns (**Fig 2A**). Next, we used the same computational tools as in the *sams-1* analysis to compare expression patterns of *P*. *aeruginosa* response genes after *set-2* RNAi.

**Fig 4:**
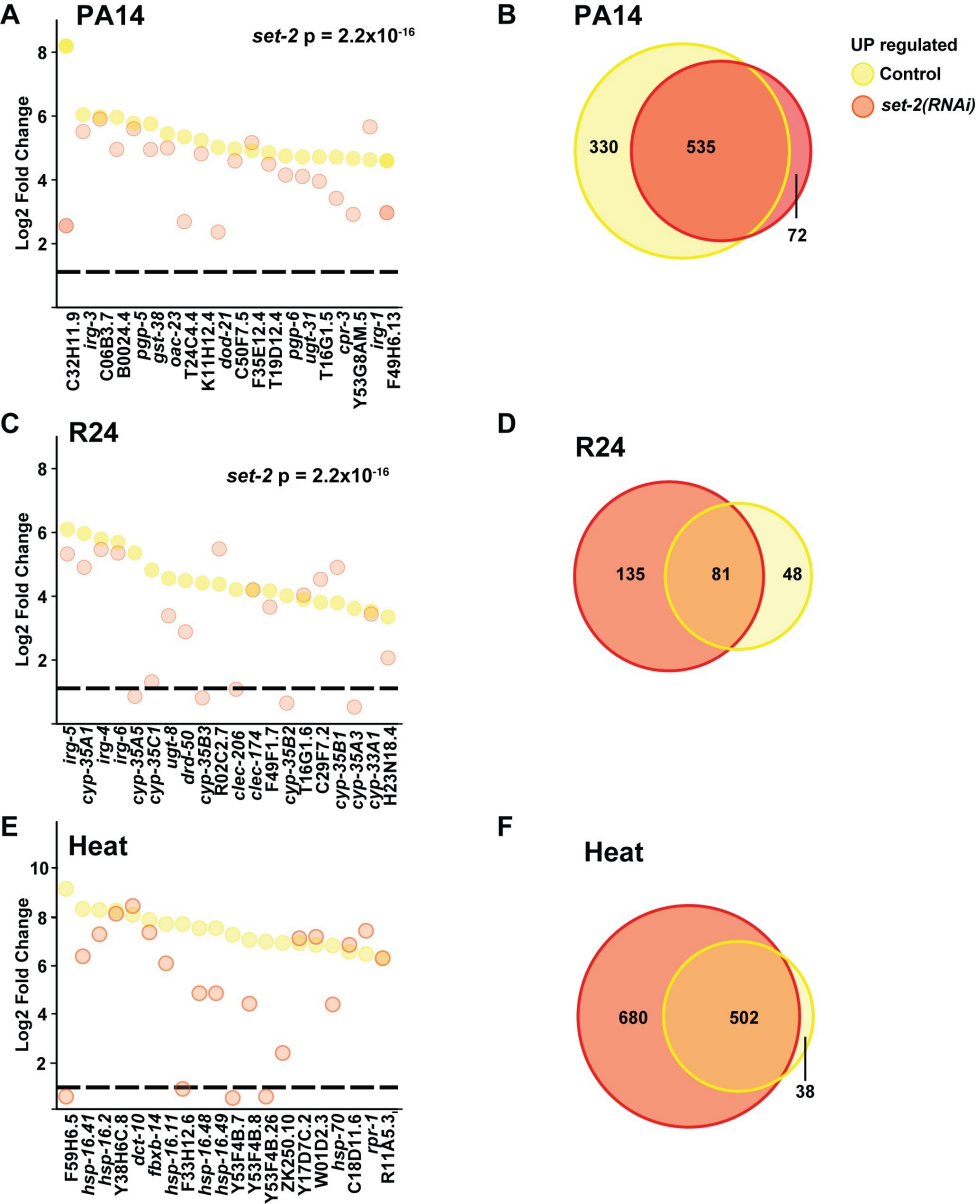
Differential transcriptional responses to a bacterial, xenotoxic and heat stress after *set-2(RNAi)*. (**A**) Strip plots show that many of the top 20 genes upregulated in response to *P*. *aeruginosa* are reduced after *set-2* RNAi. KS calculations were used to determine significance. (**B**) Venn diagrams show that the majority of genes upregulated after *set-2(RNAi)* in response to *P*. *aeruginosa* were also upregulated in controls. (**C**) Strip-plot shows that many of the top 20 genes induced by R24 in control animals are reduced after *set-2* RNAi. (**D**) Venn diagram shows that *set-2(RNAi)* animals induce many genes outside the response to R24 seen in control animals. (**E**) Strip plots demonstrate that most of the top 20 genes induced in response to heat are expressed at similar levels in *set-2* RNAi animals. (**F**) Venn diagrams show that many the majority of genes induced more than 2-fold in control animals are also upregulated after heat stress in *set-2* RNAi animals and that expression of many additional genes also increases. RNA for sequencing was isolated from control, *sams-1*, *set-2* and *set-16* RNAi as a set for each stress. Therefore, control genes in A-F are the same as in Fig 1 for *P*. *aeruginosa* and Fig 2 for R24 and heat.

Interestingly, although heat maps show that activation of bacterial-stress responsive genes are diminished after *set-2* RNAi, the effect is less severe than in *sams-1(RNAi)* animals (**Fig 2A**). Direct comparison of the 20 most highly expressed genes shows reduced expression of several genes in *set-2* animals, in line with an intermediate effect between controls and *sams-1* (**Figs 2A and 4A**) and Venn diagrams show the *P*. *aeruginosa*-responsive genes in *set-2* animals were primarily included in the control response **(Fig 4B**). Analysis of the genes two-fold downregulated in control animals shows that most of the genes were reduced at similar levels after *set-2* RNAi and were part of the same transcriptional response (**S4A and S4B Fig**). Taken together, this data suggests that *set-2* RNAi mediates part of the response to *P*. *aeruginosa* in low SAM.

Low SAM decreased the ability of *C*. *elegans* to respond transcriptionally to xenobiotic R24 (**Fig 3A–3C**). Interestingly, knockdown of *set-2* mirrored *sams-1* RNAi in some respects, but not others. Like *sams-1*, heat maps show that *set-2* RNAi limited the number of genes upregulated by more than two-fold (**Fig 3A, S3 Table**). As with the response to *P*. *aeruginosa*, *set-2* RNAi animals show reductions in several of the top 20 R24-induced genes and have diminished genome-wide expression of R24 response genes (**Fig 4C**). However, a set of 135 genes were induced in response to R24 in *set-2* RNAi animals that were not upregulated in control samples (**Fig 4D**), suggesting deregulation or expansion of the transcriptional response.

Next, we examined the response to heat stress after *set-2* RNAi and found similarities with the *sams-1* response. First, many genes induced more than twofold are similar in *set-2* RNAi and controls (**Fig 3D, S4 Table**). Second, although most of the top 20 genes upregulated in control animals were expressed after *set-2* RNAi at near normal levels (**Fig 4F**). Strikingly, many genes ectopic to the control response were induced after *set-2* RNAi (**Fig 4G**). As in the upregulated gene sets, the downregulated genes in control animals in response to heat also decreased after *set-2* RNAi. However, a large number of genes not downregulated in controls also decreased (**S5E and S5F Fig**). Thus, *set-2* appear to be important for full response to *P*. *aeruginosa* or R24, but dispensable for genes induced by heat in control animals. Interestingly, knockdown of this H3K4 methyltransferase appears to deregulate or expand the stress response to both R24 and heat.

### Differential attenuation of stress-responses after knockdown of the H3K4 methyltransferases *set-16*

**Fig 5:**
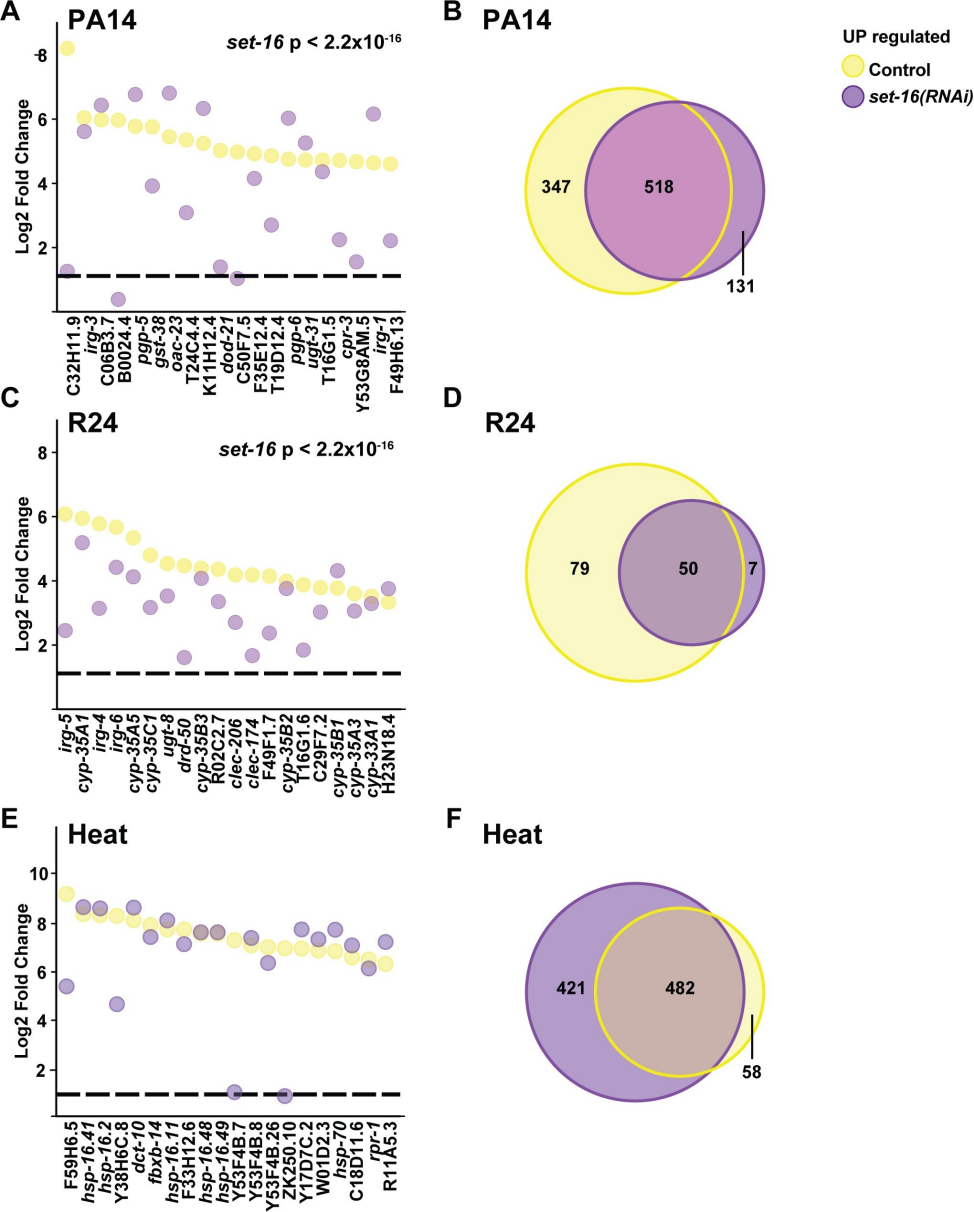
*set-16* is important for gene expression after bacterial and xenotoxic stress genes. (**A**) Strip plots show that many of the top 20 genes upregulated in response to *P*. *aeruginosa* are reduced after *set-16* RNAi. KS calculations were used to determine significance. (**B**) Venn diagrams show that the majority of genes upregulated after *set-16(RNAi)* in response to *P*. *aeruginosa* were also upregulated in controls. (**C**) Strip-plot shows that the majority of the top 20 genes induced by R24 in control animals are reduced after *set-16* RNAi. (**D**) Venn diagram shows that genes induced by more than two-fold *set-16(RNAi)* animals are also induced in controls. (**E**) Strip plots demonstrate that most of the top 20 genes induced in response to heat are expressed at similar levels in *set-16* RNAi animals. (**F**) Venn diagrams show that many the majority of genes induced more than 2-fold in control animals are also upregulated after heat stress in *set-16* RNAi animals, and that expression of many additional genes also increases. RNA for sequencing was isolated from control, *sams-1*, *set-2* and *set-16* RNAi as a set for each stress. Therefore, control genes in A-F are the same as in Fig 1 for *P*. *aeruginosa* and Fig 2 for R24 and heat.

Like *set-2*/SET1, *set-16*/MLL is important for H3K4me3 in the *C*. *elegans* intestine [8]. In addition, we identified a critical role for *set-16* in mediating *P*. *aeruginosa*-responsive gene regulation in our previous studies [8]. Therefore, we also compared bacterial, xenotoxic and heat stress induction in knockdown of *set-16* to *set-2* and *sams-1*. Confirming our previous qPCR analysis of selected *P*. *aeruginosa*-responsive genes in *set-16* RNAi animals, we found that *set-16* was broadly important for expression of genes upregulated by bacterial stress (**Fig 2A**). Many of the highest expressed genes in control animals during the *P*. *aeruginosa* response were diminished after *set-16(RNAi)* **(Fig 5A, S3 Table**). Finally, most of the genes upregulated by *P*. *aeruginosa* in *set-16* RNAi animals were also upregulated in control samples (**Fig 5B**). A proportion of the genes downregulated by *P*. *aeruginosa* in control animals were also downregulated in *set-16* RNAi animals (**S6A and S6B Fig**). Thus, *sams-1*, *set-2*, and *set-16* all appear to have critical roles in regulating genes in response to bacterial stress in *C*. *elegans*.

Responses to R24 in *set-16(RNAi)* animals largely mirrored *sams-1* knockdown but were distinct from *set-2*. Both the number of expressed genes and the levels of the highest expressed genes were significantly decreased (**Figs 3A, 5C and 5D**). Finally, the majority of genes that increased in *set-16(RNAi)* animals also increased in controls (**Fig 5D**). The majority of the genes downregulated by R24 in control animals were not similarly downregulated after *set-16* RNAi (**S6C and S6D Fig**) As in *sams-1* and *set-2* knockdown, the top twenty expressed genes in control animals were expressed similarly in heat-shocked control and *set-16(RNAi)* animals (**Fig 5E**) and *set-16(RNAi)* animals deregulated or expanded heat-stressed induced gene expression patterns compared to controls (**Fig 5F**). Like *sams-1(RNAi)* or *set-2(RNAi)*, *set-16* animals upregulated and downregulated genes whose expression were not part of the response in control animals (**Fig 5I; S5E and S5F Fig**). Taken together, our results suggest that low SAM, decreased *set-2*/SET1 and *set-16*/MLL activity all compromise bacterial stress-induced gene expression. Xenotoxic stress induced by R24 appeared to have a stronger requirement for *sams-1* or *set-16*, with many ectopic genes upregulated in the *set-2* response to R24. Finally, genes activated by heat shock appeared mostly unaffected by low SAM, decreased *set-2*/SET1 or *set-16*/MLL activity, suggesting that neither SAM or these HMTs are essential for their expression. However, each displayed a significant number of ectopic genes inductions in both up- and down-regulated gene sets. This suggests that complex regulatory interactions may lie downstream of SAM or the H3K4 tri-methylases during the heat shock response. These could include regulation of downstream transcription factors, methylation of other histone or DNA targets or methylation-independent activity of COMPASS complexes [12].

### Survival of *set-2* and *set-16* RNAi animals differs during bacterial, xenotoxic or heat stress

**Fig 6:**
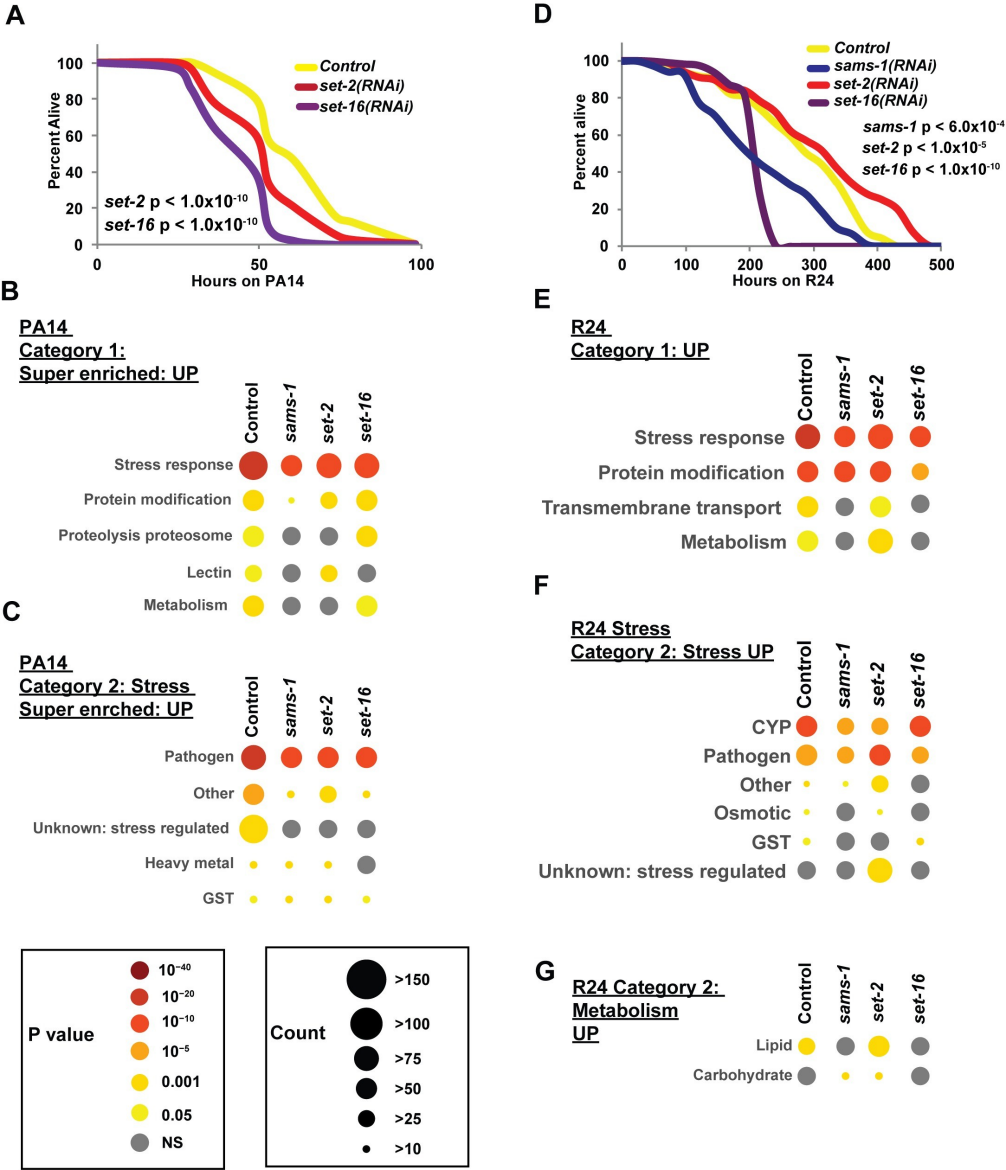
Stress-responsive and metabolic gene regulation are linked to survival after bacterial or xenotoxic stress in low SAM or H3K4 MT knockdown. (**A**) Representative Kaplan-Meier survival plot of *set-2* or *set-16* adults exposed to *P*. *aeruginosa* shows increased sensitivity to bacterial stress (data and additional statistics available in **S5 Table**). Statistical significance is shown by Log-rank test. (**B**) Bubble charts show broad category enrichment of upregulated genes determined by Worm-Cat in control, *sams-1*, *set-2* or *set-16* animals in genes changed more than 2-fold (FDR <0.01) after *P*. *aeruginosa* exposure. Specific category enrichment of stress (**C**) categories in *sams-1*, *set-2* or *set-16* animals in genes changed more than 4-fold (FDR <0.01) after *P*. *aeruginosa* exposure. Complete category enrichment data is available in **S8 Table.** (**D**) Representative Kaplan-Meier survival plot of *sams-1*, *set-2* or *set-16* RNAi adults exposed to R24 shows differential sensitivity to xenotoxic stress (data and additional statistics available in **S9 Table**). Statistical significance is shown by Log-rank test. (**E**) Bubble charts show broad category enrichment of upregulated genes determined by Worm-Cat in control, *sams-1*, *set-2* or *set-16* animals in genes changed more than 2-fold (FDR <0.01) after R24 exposure. Specific category enrichment of stress (**F**) or metabolic (**G**) categories in *sams-1*, *set-2* or *set-16* animals in genes changed more than 2-fold (FDR <0.01) after R24 exposure. Complete category enrichment data is available in **S10 Table**.

We found that *sams-1*, *set-2*, and *set-16* were all required for the transcriptional response to bacterial stress, but differentially affected the response to the xenotoxic agent R24. Moreover, although *sams-1*, *set-2*, and *set-16* were not required for heat shock gene expression, a varied but significant number of ectopic genes increased or decreased expression when knockdown animals were subjected to heat shock. Next, we sought to determine how low SAM or H3K4 HMT knockdown affected survival during each stress response. Previously, we found that *sams-1(lof)* animals had poor survival on *P*. *aeruginosa*, which was matched by attenuated expression of bacterial-stress responsive genes, and impairment H3K4me3 acquisition at bacterial-stress responsive genes after infection [8]. To determine if *set-2* or *set-16(RNAi)* animals shared this susceptibility to bacterial stress, we challenged control and knockdown animals with *P*. *aeruginosa* and determined survival rates. Concordant with the whole genome RNA sequencing data (**Figs 4A–4C and 5A–5C**), we found that both *set-2* and *set-16(RNAi)* animals had significantly reduced survival on *P*. *aeruginosa* (**Fig 6A, S5 Table**).

Our whole-genome expression analysis showed that many of the genes upregulated in control animals were part of the well-described transcriptional response to *P*. *aeruginosa* [28]. However, we also noted other gene sets that could have essential survival functions. When using GO term analysis with the Gorilla website (http://cbl-gorilla.cs.technion.ac.il/), we found that 32% of recognized genes were not associated with a GO term (**S7 Fig**). Thus, we built an annotation tool, Worm-Cat to categorize a more complete list of *C*. *elegans* genes and determine gene enrichment scores through Fisher's exact test. Worm-Cat allows assignment of broad physiological or molecular categories (i.e., stress response), and then subsequently identifies specific sub-categories (pathogen, heavy metal, etc.) (See **S6 Table for annotation table**). If genes do not have a clear physiological function or are pleiotropic, molecular functions were used. We validated this tool by comparison with GO analysis of our previously published microarray data from *sams-1* and *sbp-1(RNAi)* [8] (**S7 Table**). The most significant categories, such as stress response pathogen in *sbp-1(RNAi)* and *sams-1(RNAi)* upregulated genes or fatty acid metabolic genes in *sbp-1(RNAi)* down, or *sams-1(RNAi)* upregulated genes were identified by GO and by WORMCAT. While transcriptional regulation was identified by GO ontogeny for *sams-1(RNAi)* upregulated genes, our tool showed a breakdown showing an enrichment for nuclear hormone receptors, providing additional specificity. This tool was also able to show enrichment for regulation of 1CC genes in *sbp-1* downregulated genes, which we had previously noted [24], but were not identified by GO ontogeny. Thus, this tool increases the depth and specificity of gene function in comparison to GO term enrichment.

We used Worm-Cat to determine the major categories of genes that were changed in control, *sams-1*, *set-2* or *set-16* RNAi animals, then determined which categories matched survival patterns. In large sets of regulated genes, super enrichment can be a valuable tool for assessing the role of genes with the largest difference in gene expression patterns [34]. Therefore, we determined enrichment scores for both 2 fold and 4 fold enriched genes (**Fig 6B–6D** and **S8 Table**). Although stress-responsive genes were still enriched after *sams-1*, *set-2* or *set-16* RNAi, both the enrichment score and gene number were reduced at both the Category 1 (**Fig 6B**) and more specific Category 2 levels (**Fig 6C, S8 Table**). Surprisingly, metabolic genes were a significant fraction of genes upregulated in control animals and less enriched after *sams-1*, *set-2* or *set-16* RNAi (**Fig 6B**), correlating with poor survival of these animals. A breakdown of specific categories of metabolic genes showed that lipid metabolism was a significant category in control animals and that there were fewer genes and lower enrichment scores after *sams-1*, *set-2* or *set-16* RNAi. Interestingly, fatty acid desaturases have been linked to response of *C*. *elegans* to *P*. *aeruginosa* [25]. In addition, 1CC genes are among those downregulated by *C*. *elegans* during *P*. *aeruginosa* infection ([28], see also **S8 Table**)). Taken together, this suggests metabolic regulation may be an important part of these bacterial stress response.

R24 is a xenotoxic agent that activates both immune and detoxification responses [25–27]. We found that transcriptional responses to R24 were distinct in *sams-1*, *set-2*, and *set-16* animals, with genes ectopic to the control xenotoxic stress response increasing in *set-2* RNAi animals (**Fig 3A–3C; Fig 4D–4F; Fig 5D–5F**). To determine how *sams-1*, *set-2* and *set-16* knockdown affected survival, we treated animals with R24 and monitored death rates. We found that knockdown of *sams-1*, *set-2*, and *set-16* had differential susceptibility to R24-mediated toxicity. First, we found that concordant with the reduced expression of xenotoxic agent-response genes, *sams-1* and *set-16* had poor survival rates, with *set-16* animals showing a particularly sharp decline (**Fig 6E, S9 Table**). Knockdown of *set-2*, however, did not decrease survival. Next, we used Worm-Cat to identify gene categories that might correlate with sensitivity to the xenotoxic agent in *sams-1* and *set-16* animals, or survival in the *set-2* cohort. First, we noticed that as expected, stress-response genes were the most enriched category in control animals, with fewer genes in the sensitive *sams-1* or *set-16* animals (**Fig 6F, S10 Table**). Breakdown of stress categories shows that R24-induced genes are enriched for cytochrome P450 genes and pathogen response genes (**Fig 6G**), as expected for R24 [25–27]. However, the knockdowns most sensitive to R24 (*sams-1* and *set-16*) differed slightly in their stress response profiles. After RNAi of *sams-1*, both CYP450 and pathogen response gene categories loose enrichment (**Fig 6G, S10 Table**). However, *set-16* lost enrichment only within the pathogen category (**Fig 6G, S10 Table**), suggesting that genes within the pathogen response category may be important for survival on R24. Supporting this notion, *set-2*, which survived normally, lost CYP450 enrichment but retained genes in the pathogen response category (**Fig 6G, S10 Table**). We also noted that metabolic categories were also limited in the sensitive strains. Metabolic genes, particularly in the lipid metabolism category were significantly enriched in Control and *set-2* RNAi animals after R24 treatment, but enrichment scores failed significance after *sams-1* or *set-16* RNAi (**Fig 6F and 6H, S10 Table**). This suggests that as in the bacterial stress response, rewiring metabolic genes correlates with stress survival. Notably, an RNAi screen for R24-dependent regulators of the innate immune response gene *irg-4* identified multiple genes involved in fatty acid synthesis [25]. Finally, we found that genes deregulated in *set-2(RNAi)* animals were enriched for genes activated by multiple stresses (**Fig 6F, S10 Table**). However, since these genes have no other functional classification, their importance of this gene set is unclear.

### SAM and the H3K4 methyltransferases *set-2* and *set-16* are differently required for survival during heat stress

**Fig 7:**
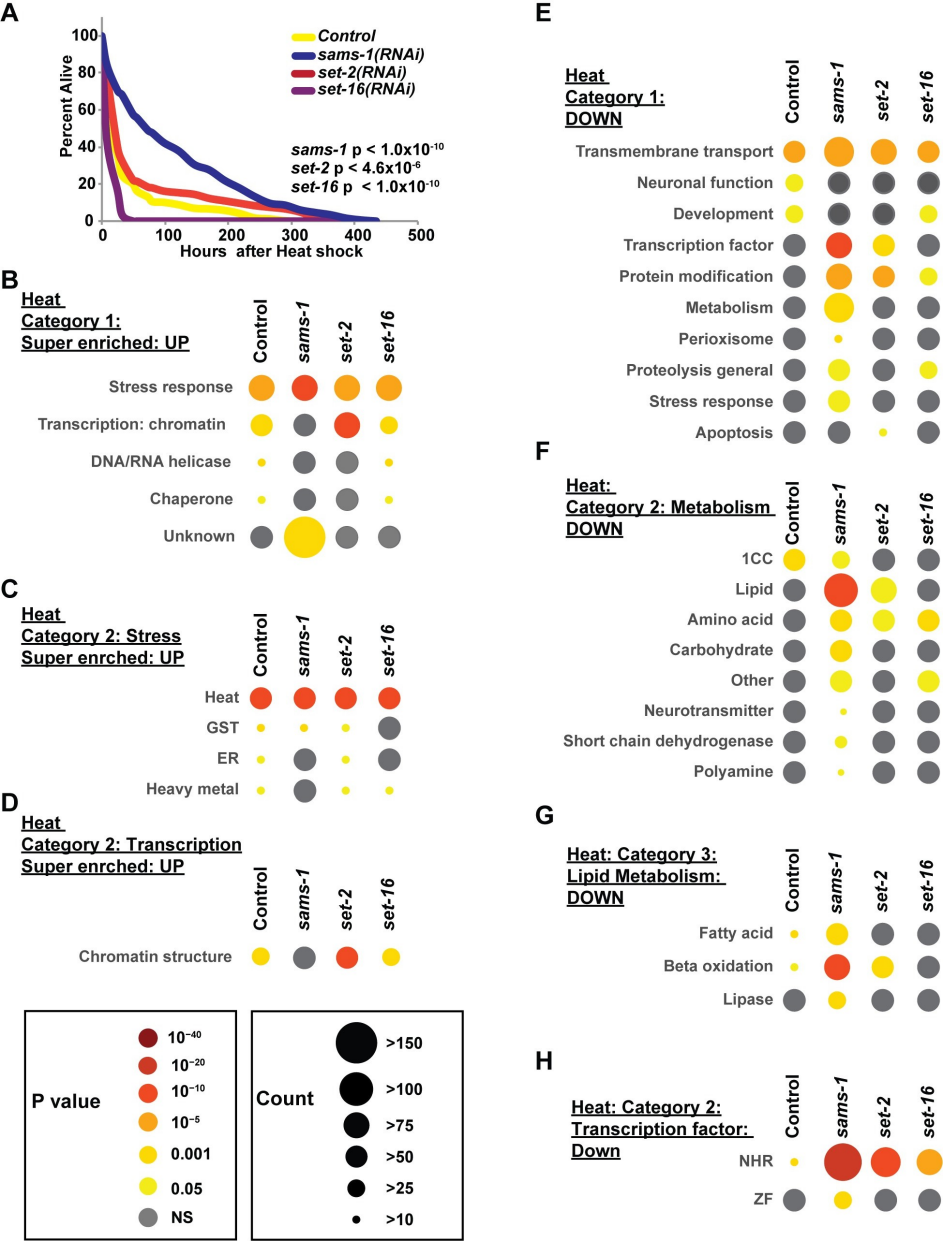
Down-regulation of metabolic gene regulation is linked to survival after heat stress in low SAM or H3K4 MT knockdown. (**A**) Representative Kaplan-Meier survival plot of control, *sams-1*, *set-2* or *set-16* adults exposed to heat shows increased sensitivity to heat stress (data and additional statistics available in **S11 Table**). Statistical significance is shown by Log-rank test. For additional replicates. (**B**) Bubble charts show broad category enrichment of upregulated genes determined by Worm-Cat in control, *sams-1*, *set-2* or *set-16* animals in genes changed more than 4-fold (FDR <0.01) after heat exposure. Specific category enrichment of stress (**C**) or transcription (**D**) categories in control, *sams-1*, *set-2* or *set-16* animals in genes changed more than 4-fold (FDR <0.01) after heat exposure. (**E**) Bubble charts show broad category enrichment of downregulated genes in control, *sams-1*, *set-2* or *set-16* animals in genes changed more than 2-fold (FDR <0.01) after heat exposure. Specific category enrichment of metabolism (**F**), lipid metabolism (**G**) or transcription (**H**) categories in control, *sams-1*, *set-2* or *set-16* animals in genes changed more than 4-fold (FDR <0.01) after heat exposure. Complete category enrichment data is available in **S12 Table**.

Transcription of heat shock genes in response to high temperature is controlled by shifting RNA pol II from a paused to the elongating form at heat shock response genes [32]. To provide a comparison to bacterial or xenotoxin-induced stress, we compared heat-shock responsive transcription in low SAM or after knockdown of the *set-2*/SET1 or *set-16*/MLL methyltransferases to transcriptional changes occurring after bacterial or xenotoxic stress responses. Strikingly, we found that many genes ectopic to the control heat shock response were activated or repressed after *sams-1*, *set-2* or *set-16* RNAi (**Figs 3D–3F, 4H–4J and 5H–5J**). Next, we performed survival assays to determine if these gene expression changes altered survival of these animals during stress. Unlike the bacterial stress response, *sams-1*, *set-2*, and *set-16* all had distinct survival curves. First, *sams-1* animals were markedly resistant during the first half of the assay, with the survival percentage at the assay midpoint more than twice that of control animals (**Fig 7A, S11 Table**). The endpoint of the assay, however, was close to controls. Second, as in R24 assays, *set-2* animals survived most similar to controls, although p values showed a significant difference (**Figs 6A and 7A, S11 Table**). Finally, *set-16* RNAi caused an extreme sensitivity to heat stress (**Fig 7A, S11 Table**), similar to *P*. *aeruginosa* and R24 responses.

To determine if categories of genes expressed in the *sams-1*, *set-2* or *set-16* RNAi animals correlated with the differential survival, we used Worm-Cat to survey the enriched and super enriched heat responsive genes. As expected from our initial analysis, significant numbers of stress-responsive genes were enriched in the upregulated *sams-1*, *set-2* and *set-16* RNAi cohorts (**Fig 7B and 7C, S12 Table**). Genes in chromatin structure were also enriched in all but *sams-1* RNAi animals (**Fig 7B and 7D, S12 Table**). However, none of these category clusters correlated with survival. Next, we examined the categories enriched in the genes downregulated during the heat shock response (**Fig 7E–7G; S12 Table**). While expression of canonical heat shock genes did not correlate with survival, we found enrichment in other functional categories. There were several categories of enriched genes (transmembrane transport, proteolysis, and protein modification) among the *sams-1*, *set-2* or *set-16* RNAi animals (**Fig 7E, S12 Table**). However, two categories correlated best with survival: metabolism and transcription factors. Strikingly, metabolism was only enriched as a downregulated category in *sams-1(RNAi)* animals during heat shock, with the majority of these genes in anabolic pathways such as lipid and amino acid metabolism (**Fig 7E and 7F; S12 Table**). This is distinct from PC-dependent effects from *sams-1* in basal conditions, where fatty acid genes are activated (see **S7 Table, S12 Table**). *C*. *elegans* contains a major expansion of nuclear hormone receptors, many of which are thought to regulate metabolic processes [35]. Intriguingly, we also observed reduced NHR gene expression *sams-1(RNAi)* animals (**Fig 7G, S12 Table**), concomitant with the loss of metabolic gene expression. Finally, we find that regulation of metabolic gene expression also correlates with survival in heat stress, as it did in our bacterial or xenotoxic stress assays, suggesting metabolic flexibility may be a common effector in stress response survival (**S8 Fig**).

## Discussion

Metabolites that contribute to cellular regulatory functions, such as the methyl donor SAM, could be predicted to have broad effects on transcription. Indeed, SAM has been proposed as a link between nutrition and regulation of the 1CC to transgenerational epigenetic effects [5]. However, work from several labs across multiple systems has shown that SAM has surprisingly specific effects on histone methylation, reducing or increasing H3K4me3 as levels fall or rise [6–9]. Since H3K4me3 is tightly associated with start sites of actively transcribed genes, this suggests SAM may also have a critical role in acute gene regulatory events. Recently, the Locasale lab has shown that H3K4me3 peak breadth is sensitive to methionine levels in mouse liver and human cancer cells, strengthening the connections between SAM and H3K4me3 *in vivo* [11].

In this study, we have defined a role for SAM in the regulation of two stress responses, bacterial and xenotoxic stress, and found that it is necessary for induction of specific response genes, as well as for survival. This link between 1-carbon metabolism and stress responses has important implications for how organisms can respond to stress when metabolically challenged. Interestingly, while heat shock genes were expressed independently of SAM, many other genes had altered expression patterns in heat shocked *sams-1(RNAi)* animals which survived better than wild type. This suggests multiple independently regulated modules can contribute to survival. While the expression of heat-shock response genes at control levels in *sams-1(RNAi)* animals suggest these are regulated independently of SAM, the ectopically regulated genes could respond to SAM from direct or indirect mechanisms. For example, Labbadia and Morimoto have recently shown that in *C*. *elegans*, non-cell autonomous mechanisms linked to repressive H3K27me3 limit stress responses when reproduction starts [36]. Thus, this ectopic gene activation in *sams-1(RNAi)* animals could result from changes in repressive methylation on other histones, on DNA or through other indirect effects.

Our Worm-Cat annotation tool has shown that downregulation of two classes of genes correlates with survival of *sams-1* RNAi animals under heat shock: metabolic genes (amino acid, lipid and beta-oxidation) and nuclear hormone receptors. We hypothesize that downregulating these categories pauses anabolic processes and allows a survival advantage for *sams-1* animals as they respond to heat stress. Interestingly, fatty acid synthesis genes are upregulated after *sams-1* RNAi in basal conditions, as changes in PC levels activate the lipogenic transcription factor SBP-1 [24]. As in our study of low SAM on *C*. *elegans* on normal laboratory diet of *E*. *coli* or *P*. *aeruginosa* where we found differential effects on activation or repression of pathogen response genes [8], this suggest the effects of low SAM may differ in distinct stress or nutritional conditions. Future metabolomic studies will be important for how these changes in metabolic gene expression are linked to survival during heat stress. Nuclear hormone receptors are common regulators of metabolic genes [35], and while direct relationships between these nuclear hormone receptors and the metabolic genes identified in our study are not yet discernable, it is intriguing that both classes of these genes are downregulated in the surviving animals. Thus, low SAM may have both direct and indirect effects that influence gene expression and survival during stress responses.

SAM is utilized by HMTs such as *set-2*/SET1 or *set-16*/MLL to produce methyl marks such as H3K4me3. Intriguingly, KTM2s are among the most sensitive HMTs to SAM levels [5]. SET1 is the single H3K4me3 in yeast, and thus essential for all H3K4 methylation [12]. Neither *set1* or H3K4 tri-methylation are essential for viability under standard conditions [14]. However, *set1* appears to function to limit the expression of ribosomal genes during the response to diamide [15]. The mammalian methyltransferase family is complex with seven H3K4 methyltransferases that differ in specificity for mono, di or trimethylation [12]. However, it has been difficult to assign specific biological functions. In *C*. *elegans* where the KTM2 family is simpler, we have found that *set-2* or *set-16* RNAi mirrors some of the effects of low SAM, reducing transcriptional responses to multiple stresses. However, *set-2*/SET1 and *set-16*/MLL appeared to have distinct functional profiles during these stress responses. *set-2*/SET1 RNAi is similar to low SAM in response to bacterial stress. Some SAM-dependent *P*. *aeruginosa* responsive genes are also limited in expression after *set-2* RNAi, and *set-2* animals survive poorly. Our previous analysis of the *P*. *aeruginosa* response in *set-2* and *set-16* animals suggested that *set-2* may have a more limited role [8]. The present whole genome study also bears out an essential role for *set-16* in *P*. *aeruginosa*-responsive transcription, but notably, *set-2* animals are also survived poorly on *P*. *aeruginosa*, suggesting that critical genes are limited in both cases. However, *set-2* appears less critical for some genes in the detoxification response to R24. Survival is close to wild-type, and intriguingly, metabolic genes related to lipid synthesis are upregulated, distinct from the control response. Like the response to low SAM during heat shock, *set-2* RNAi did not limit expression of genes induced by heat shock in control animals while many genes were de-repressed or ectopically regulated outside the response in controls. As with R24, *set-2* RNAi animals survived similar to controls during heat stress. Therefore changes in gene expression did not impact these biological functions. *set-2* also has intriguing functions during lifespan regulation in *C*. *elegans*. Greer et al. showed transgenerational effects on lifespan in *set-2* mutants, and another study from the Brunet lab suggested that *set-2* and another H3K4 HMT (*ash-2*) linked lipid synthesis and lifespan regulation [21]. They found that *ash-2* was important for non-cell autonomous germline to intestine regulation of these processes [21]. However, the role of *set-2* in direct regulation was less clear. Notably, in our study, although lipid biosynthetic genes were not changed in *set-2* animals at the L4/young adult time point, many of these genes did increase upon R24 treatment. Taken together, this suggests *set-2* may impact the regulation of lipid synthesis genes at different points during *C*. *elegans* lifespan or during specific stress responses.

*set-16*/MLL, on the other hand, was essential for survival in each of the stresses we tested. Transcriptional responses to bacterial stress and R24 were attenuated, similar to low SAM. *sams-1* and *set-16* RNAi animals both survived poorly on *P*. *aeruginosa* and following exposure to R24. However, the *set-16* RNAi animals were particularly sensitive to R24. Interestingly, *set-16* animals were more deficient in activating pathogen response than CYP in response to R24 suggesting that pathogen response genes may be essential for survival. Like *sams-1* and *set-2* RNAi, heat stress of *set-16(RNAi)* animals produced similar activation to control in the top 20 genes, in addition to ectopic activation or derepression of many other genes. However, this did not enhance survival. Taken together, this suggests that *set-16* has a distinct role in survival during diverse stress responses.

During a stress response, many genes must be coordinately regulated downstream of specific signaling pathways. For example, pathogenic stress may be sensed by activation of Toll-like receptors in mammals and *Drosophila* [37] or by translational attenuation in *C*. *elegans* [38]. These signals are carried through stress-specific transcription factors that activate protective genes. Along with these direct regulatory pathways, the chromatin environment must be permissive. It is intriguing that a metabolic pathway producing the methyl donor SAM and the H3K4 methyltransferases *set-2* and *set-16* are critical to enable transcriptional responses to acute stress. This suggests that 1CC status could influence how cells or organisms could respond to outside insults. The Halsted lab, using a micropig model of alcoholic fatty liver disease, has found that dietary limitation of methyl donors markedly decreases the time for development of liver injury [39, 40], thus, we suggest that low SAM could exacerbate disease progressing by limiting the ability of a tissue to respond to additional stress. This could increase the severity or the progression of a disease by limiting cellular defensive responses. Finally, other metabolites such as Acetyl CoA and NAD+ also influence gene regulation [3]. By having multiple metabolic pathways influencing histone modification and gene regulation, cells might finely tune transcription to diverse nutritional signals providing templates for specific metabolic states.

## Materials and methods

### *C*. *elegans* culture, RNAi and stress applications

*C*. *elegans* (N2) were cultured using standard laboratory conditions on *E*. *coli* OP50. Adults were bleached onto RNAi plates for control (L4440), *sams-1*, *set-2* or *set-16* and allowed to develop to the L4 to young adult transition before stresses were applied. For bacterial stress RNA preparations, nematodes were placed on *E*. *coli* or *P*. *aeruginosa* plates for 6 hours. For xenotoxic stress applications animals were placed on DMSO or 70 uM R24 plates for 18 hours. For heat stress applications, animals were raised at 15°C from hatching then at the L4/young adult transition replicate plates were placed at 15°C or 37°C for 1 hour. After each stress, animals were washed off the plates with S-basal, then pellets frozen at -80°C. RNA was prepared as in Ding, et al. 2015 [8]. For survival assays, animals remained on plates until all nematodes were dead. Exposure to *Pseudomonas* or R24 continued for the life of the animals. Exposure to heat occurred for 120 minutes, then animals were kept at 20°C for the remainder of the assay. Dead animals were identified by gentle prodding and removed each day. Kaplan-Meir curves were generated with OASIS [41].

### Antibodies and immunofluorescence

Immunofluorescence was performed as in Ding et al. for H3K4me3 staining. For mono or di methyl staining, animals were fixed in 1% paraformaldehyde and permeabilized in cold 100% methanol before proceeding with the remainder of the protocol used in Ding, et al. 2015. Antibodies used were: Tri-Methyl-Histone H3 (Lys4) Rabbit mAb #9751 (Cell Signaling), Abcam Anti-Histone H3 (di methyl K4) antibody—ChIP Grade (ab7766) (Abcam) and Anti-Histone H3 (mono methyl K4) antibody—ChIP Grade (ab889) (Abcam).

### RNA sequencing and data analysis

RNA for deep sequencing was purified by Qiagen RNA easy. Duplicate samples were sent for library construction and sequencing at BGI (China). Raw sequencing reads were processed using an in-house RNA-Seq data processing software Dolphin at University of Massachusetts Medical School. The raw read pairs first were aligned to *C*. *elegans* reference genome with ws245 annotation. The RSEM method was used to quantify the expression levels of genes (Li & Dewey, 2011, PMID: 21816040).

### Data accessibility

All RNA sequencing data is available at the Gene expression ominibus, accession numbers, GSE121511, GSE121509, GSE121510.

### Computational methods

Graphing for scatter and strip plots, Venn diagrams and bubble charts was done in R. The ontogeny category tool (Worm-Cat) consists of three parts. First, over 16,000 *C*. *elegans* genes were annotated; first by physiological role, then by molecular function. Categories contain up to three levels, for example, Proteolysis Proteasome: E3: F-box could appear as Proteolysis Proteasome in the broad Category 1 or as Proteolysis Proteasome: E3 or Proteolysis Proteasome: E3: F-box in the more specific categories 2 and 3. Genes with broad physiological functions (e.g., *ama-1*, RNA polymerase II large subunit) were retained in molecular function categories. Phenotype data from alleles or RNAi were used to annotate physiological role if corroborated in two or more different assays. In addition, genes with no other function whose expression was changed by at least two of these stresses (Methylmercury, tunicamycin, rotenone, cadmium, ethanol, D-glucose) were placed in the category: Stress response: regulated by multiple stresses. Annotations were applied to genes regulated in each condition, then statistical significance of category enrichment determined by Fisher’s exact test with a p-value of < 0.05 used to determine significance.

## Supporting information

S1 Fig Distinct patterns of gene downregulation in *sams-1(RNAi)* animals during stress. Venn diagrams comparing stress responsive gene expression in control (**A**), *sams-1* (**B**), *set-2* (**C**), and *set-16* RNAi downregulated genes (**D**). Downregulated genes were defined as decreased by 2 or more fold with an FDR of less than 0.01 in each of the stresses.
(TIF)

S2 Fig Gene expression changes after *sams-1* RNAi are largely PC dependent. (**A**) Schematic diagraming the linkages between SAM usage in H3K4me3 methylation and methylation of PC. (**B**) Scatter plot of RNA sequencing changes showing genes significantly upregulated (changed by more than two-fold with an FDR of <0.01); red is up, blue is down. (**C**) Venn diagram of *sams-1(RNAi)* microarray data from Ding, et al. 2015 comparing genes upregulated in *sams-1(RNAi)* (salmon) with those rescued by choline (yellow). (**D**) Immunofluorescence comparing H3K4me3 levels in low SAM (*sams-1 RNAi*) and in *pmt-2*(RNAi) intestinal cells, which reduce PC through SAM independent pathways [42]. (**E**) Immunofluorescence comparing mono and di-methylation of H3K4 in *sams-1(RNAi)* intestinal nuclei.
(TIF)

S3 Fig Role of *sams-1* in genes downregulated during bacterial, xenotoxic or heat stress. Strip plots of the lowest 20 expressed genes (**A, C, E**) and Venn diagrams (**B, D, F**) comparing control (yellow) and *sams-1(RNAi)* (blue) animals after treatment with *P*. *aeruginosa* (**A, B**), R24 (**C, D**) or heat stress (**E, F**).
(TIF)

S4 Fig Few significant changes in gene expression occur after *set-2* or *set-16* RNAi in basal conditions. Scatter plot showing significantly up (red) or down (blue) regulated genes after *set-2* (**A**) or *set-16* (**B**) RNAi.
(TIF)

S5 Fig Role of *set-2* in genes downregulated during bacterial, xenotoxic or heat stress. Strip plots of the lowest 20 expressed genes (**A, C, E**) and Venn diagrams (**B, D, F**) comparing control (yellow) and *set-2(RNAi)* (orange) animals after treatment with *P*. *aeruginosa* (**A, B**), R24 (**C, D**) or heat stress (**E, F**). RNA for sequencing was isolated from control, *sams-1*, *set-2* and *set-16* RNAi as a set for each stress. Therefore, control genes in A-F are the same as in **S3 Fig** for each stress.
(TIF)

S6 Fig Role of *set-16* in genes downregulated during bacterial, xenotoxic or heat stress. Strip plots of the lowest 20 expressed genes (**A, C, E**) and Venn diagrams (**B, D, F**) comparing control (yellow) and *set-16(RNAi)* (purple) animals after treatment with *P*. *aeruginosa* (**A, B**), R24 (**C, D**) or heat stress (**E, F**). RNA for sequencing was isolated from control, *sams-1*, *set-2* and *set-16* RNAi as a set for each stress. Therefore, control genes in A-F are the same as in **S3 Fig** for each stress.
(TIF)

S7 Fig Screen shot from the GoRilla GO term analysis website (http://cbl-gorilla.cs.technion.ac.il/), where we entered a regulated gene set of 1,062 genes and a baseline gene set (annotated genes from S6 Table) showing that 37% of recognized genes were not associated with GO terms. (TIF)

S8 Fig Schematic showing model that SAM is important for stress-induced gene expression. (TIF)

S1 Table Table showing genes regulated in basal conditions by *sams-1* (tabs 1–3), *set-2* (tabs 4–6) and *set-16* (tabs 7–9). (XLSX)

S2 Table Table showing *P*. *aeruginosa* regulated genes (tab1: control up, tab2: *sams-1* up; tab3: *set-2* up; tab4: *set-16* up; tab5: control down; tab6: *sams-1* down; tab5: *set-2* down; tab6: *set-16* down). Genes appearing in Troemel, et al. [28] are designated.
(XLSX)

S3 Table Table showing R24 regulated genes (tab1: control up, tab2: *sams-1* up; tab3: *set-2* up; tab4: *set-16* up; tab5: control down; tab6: *sams-1* down; tab5: *set-2* down; tab6: *set-16* down). (XLSX)

S4 Table Table showing Heat shock regulated genes (tab1: control up, tab2: *sams-1* up; tab3: *set-2* up; tab4: *set-16* up; tab5: control down; tab6: *sams-1* down; tab5: *set-2* down; tab6: *set-16* down). (XLSX)

S5 Table Table showing representative *P*. *aeruginosa* survival data. Statistics generated by OASIS (https://sbi.postech.ac.kr/oasis/)[41].
(XLSX)

S6 Table Table of all annotated genes with Category listing. Annotation was generated by assigning physiological function, then molecular function defined using homology, GO ontogeny and phenotype.
(XLSX)

S7 Table Tables comparing Worm-Cat (tabs: *sbp-1* UP, *sbp-1* DOWN, *sams-1* UP, *sams-1* DOWN) to GO terms (tabs: GO *sbp-1* UP, GO *sbp-1* DOWN, GO *sams-1* UP, GO *sbp-1* DOWN) for previously published data from microarrays from genes changed 2 fold with a p-value of < 0.05 from *sams-1(RNAi)* or *sbp-1(RNAi)* animals [8]. GO term categories that are also represented in our tool are highlighted in yellow.
(XLSX)

S8 Table Tables containing PA14 regulated gene category statistics for Control, *sams-1*, *set-2* and *set-16* RNAi (tabs show for all genes, broad, stress, metabolism for up-regulated and down-regulated genes). P values generated by Fisher's exact test, significance defined as lower than 0.05. The total number of genes in categories listed in parenthesis (Column A). Pink color shows categories with a significant change in any of the RNAi animals. NS is not significant. Tabs for both enriched (2.0 fold) and super enriched (4.0 fold) are included.
(XLSX)

S9 Table Table showing representative R24 survival data. Statistics generated by OASIS (https://sbi.postech.ac.kr/oasis/) [41].
(XLSX)

S10 Table Tables containing R24 regulated gene category statistics for *Control*, *sams-1*, *set-2* and *set-16* RNAi (tabs show for all genes, broad, stress, metabolism for up-regulated and down-regulated genes). P values generated by Fisher's exact test, significance defined as lower than 0.05. The total number of genes in categories listed in parenthesis (Column A). Pink color shows categories with a significant change in any of the RNAi animals. NS is not significant.
(XLSX)

S11 Table Table showing representative heat survival data. Statistics generated by OASIS (https://sbi.postech.ac.kr/oasis/) [41].
(XLSX)

S12 Table Tables containing heat regulated gene category statistics for *Control*, *sams-1*, *set-2* and *set-16* RNAi (tabs show for all genes, broad, stress, metabolism for up-regulated and down-regulated genes). P values generated by Fisher's exact test, significance defined as lower than 0.05. The total number of genes in categories listed in parenthesis (Column A). Pink color shows categories with a significant change in any of the RNAi animals. NS is not significant. Tabs for both enriched (2.0 fold) and super enriched (4.0 fold) are included.
(XLSX)
