# Supplementary figures and images for: Stress-responsive and metabolic gene regulation are altered in low S-adenosylmethionine
Source: PLoS Genet. 2018 Nov 28;14(11):e1007812. doi: 10.1371/journal.pgen.1007812 (PMC6287882; doi:10.1371/journal.pgen.1007812)

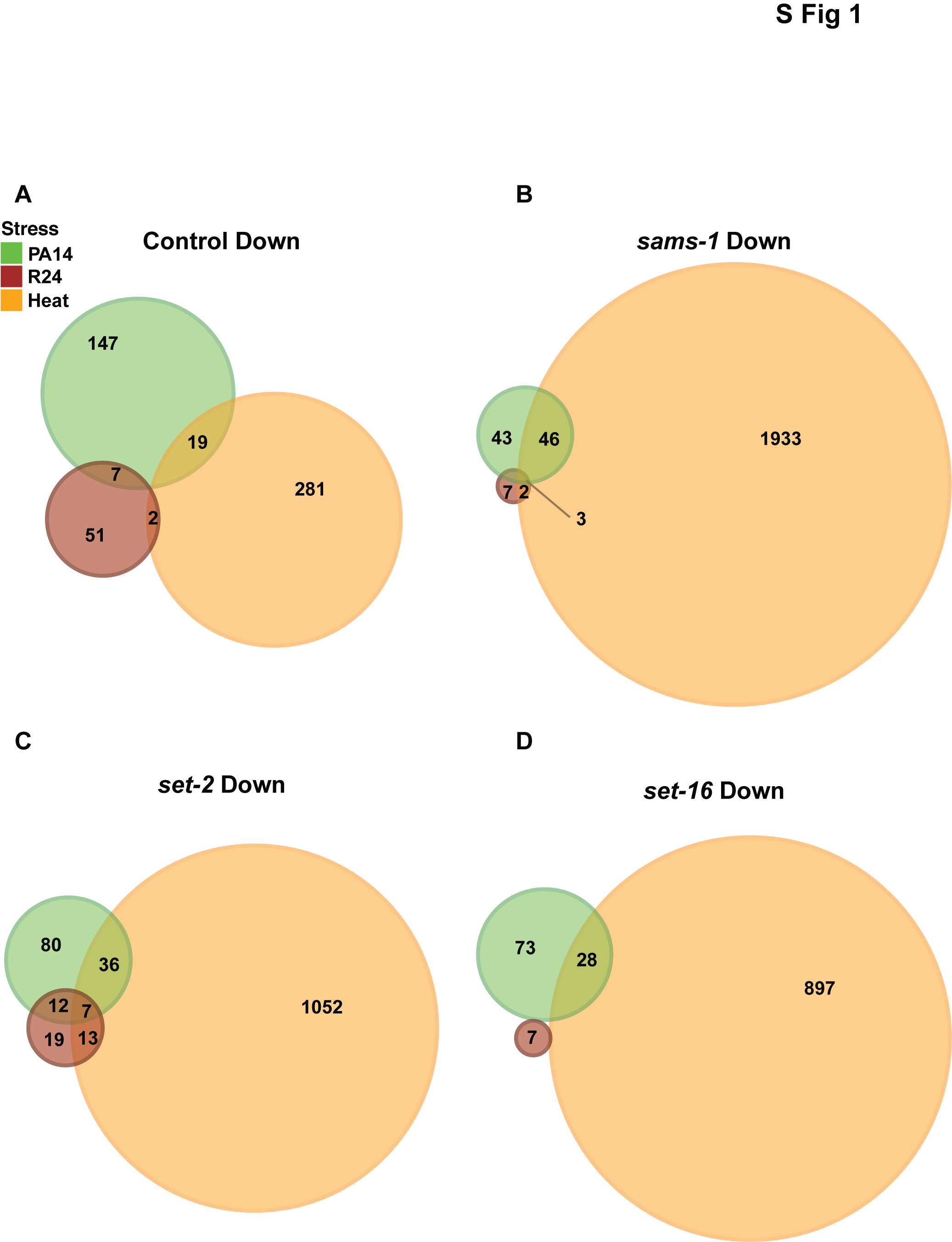

Supplement: S1 Fig — Venn diagrams comparing stress responsive gene expression in control (A), sams-1 (B), set-2 (C), and set-16 RNAi downregulated genes (D). Downregulated genes were defined as decreased by 2 or more fold with an FDR of less than 0.01 in each of the stresses. (TIF) [file pgen.1007812.s001.tif]

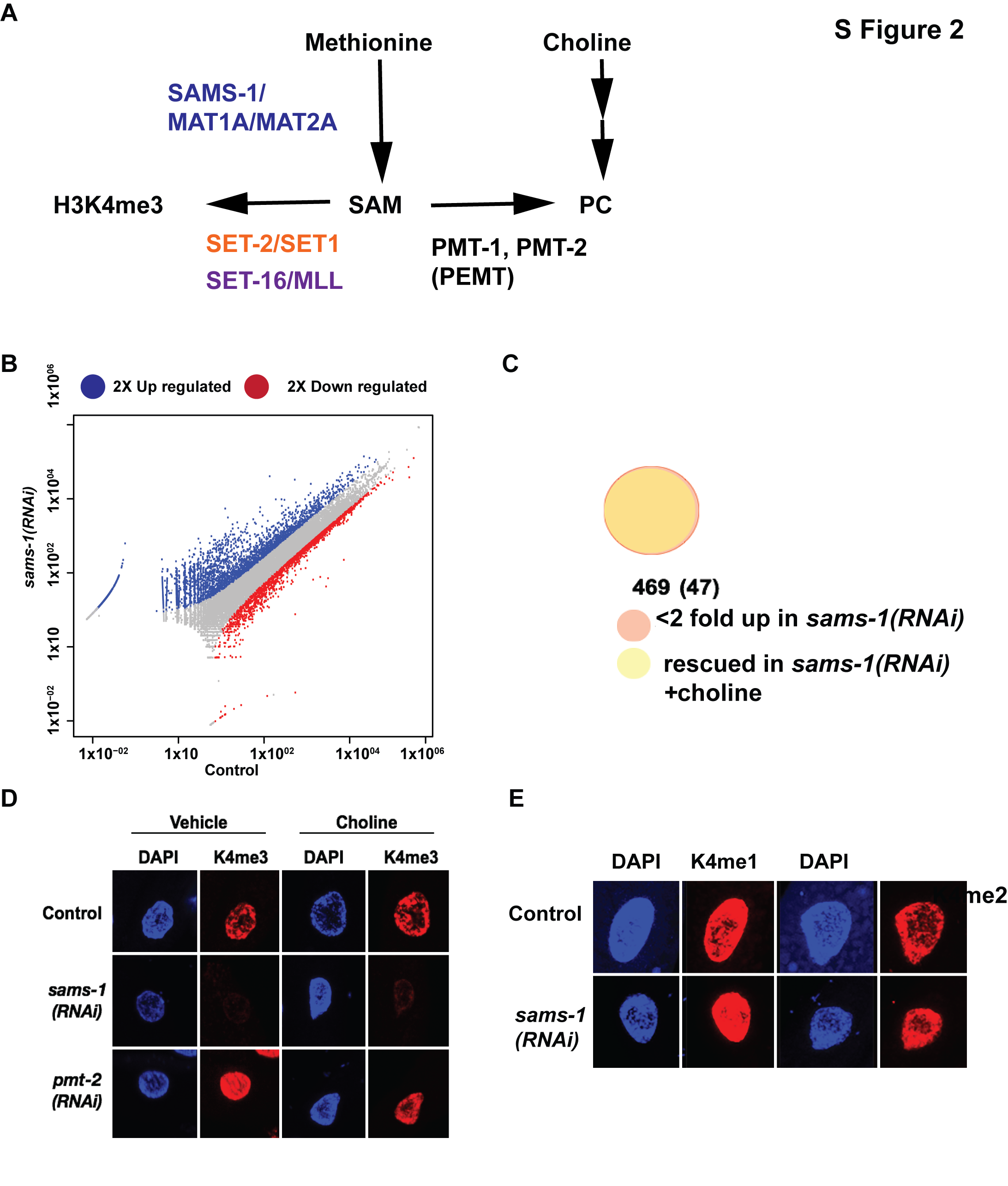

Supplement: S2 Fig — (A) Schematic diagraming the linkages between SAM usage in H3K4me3 methylation and methylation of PC. (B) Scatter plot of RNA sequencing changes showing genes significantly upregulated (changed by more than two-fold with an FDR of <0.01); red is up, blue is down. (C) Venn diagram of sams-1(RNAi) microarray data from Ding, et al. 2015 comparing genes upregulated in sams-1(RNAi) (salmon) with those rescued by choline (yellow). (D) Immunofluorescence comparing H3K4me3 levels in low SAM (sams-1 RNAi) and in pmt-2(RNAi) intestinal cells, which reduce PC through SAM independent pathways [42]. (E) Immunofluorescence comparing mono and di-methylation of H3K4 in sams-1(RNAi) intestinal nuclei. (TIF) [file pgen.1007812.s002.tif]

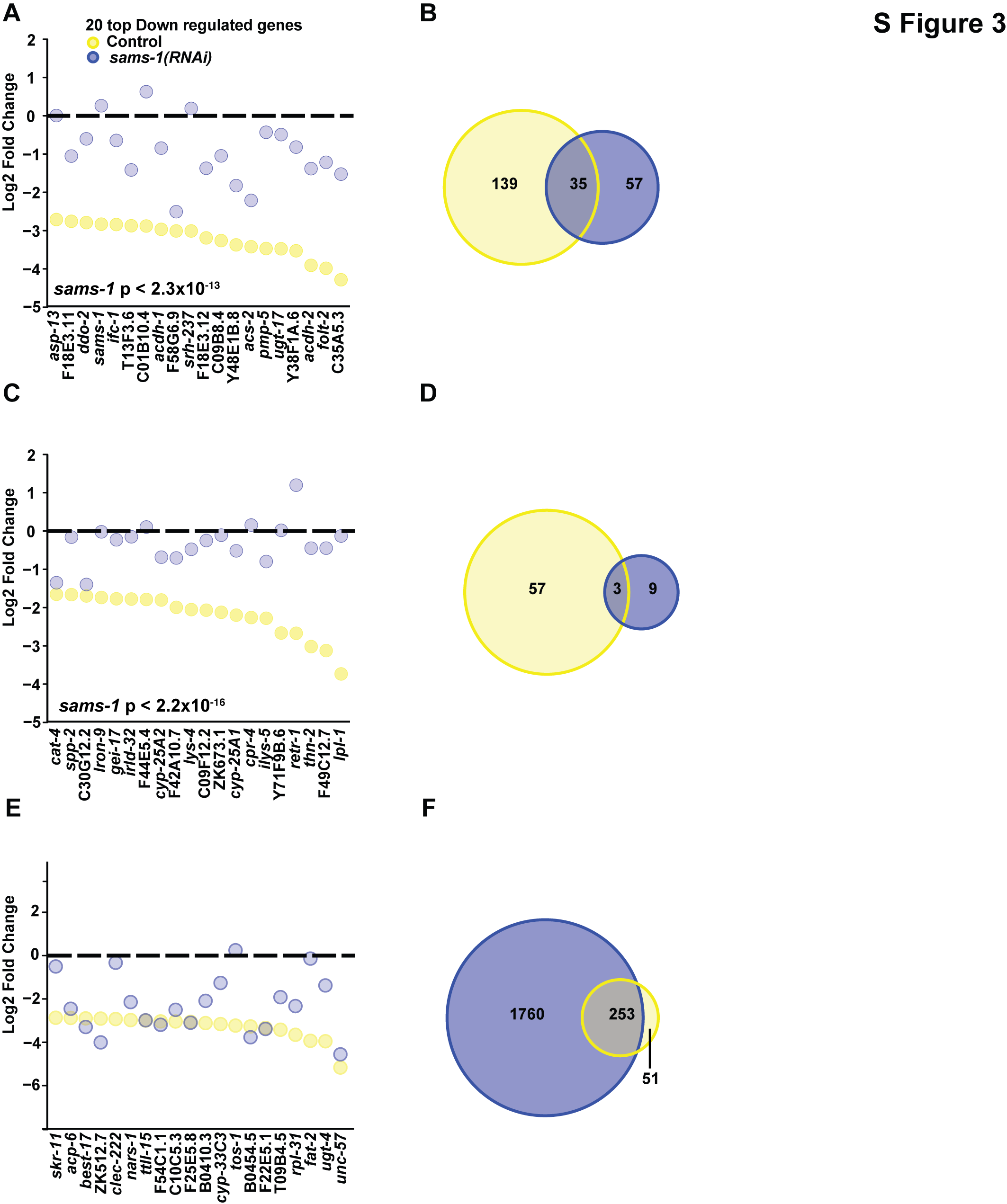

Supplement: S3 Fig — Strip plots of the lowest 20 expressed genes (A, C, E) and Venn diagrams (B, D, F) comparing control (yellow) and sams-1(RNAi) (blue) animals after treatment with P. aeruginosa (A, B), R24 (C, D) or heat stress (E, F). (TIF) [file pgen.1007812.s003.tif]

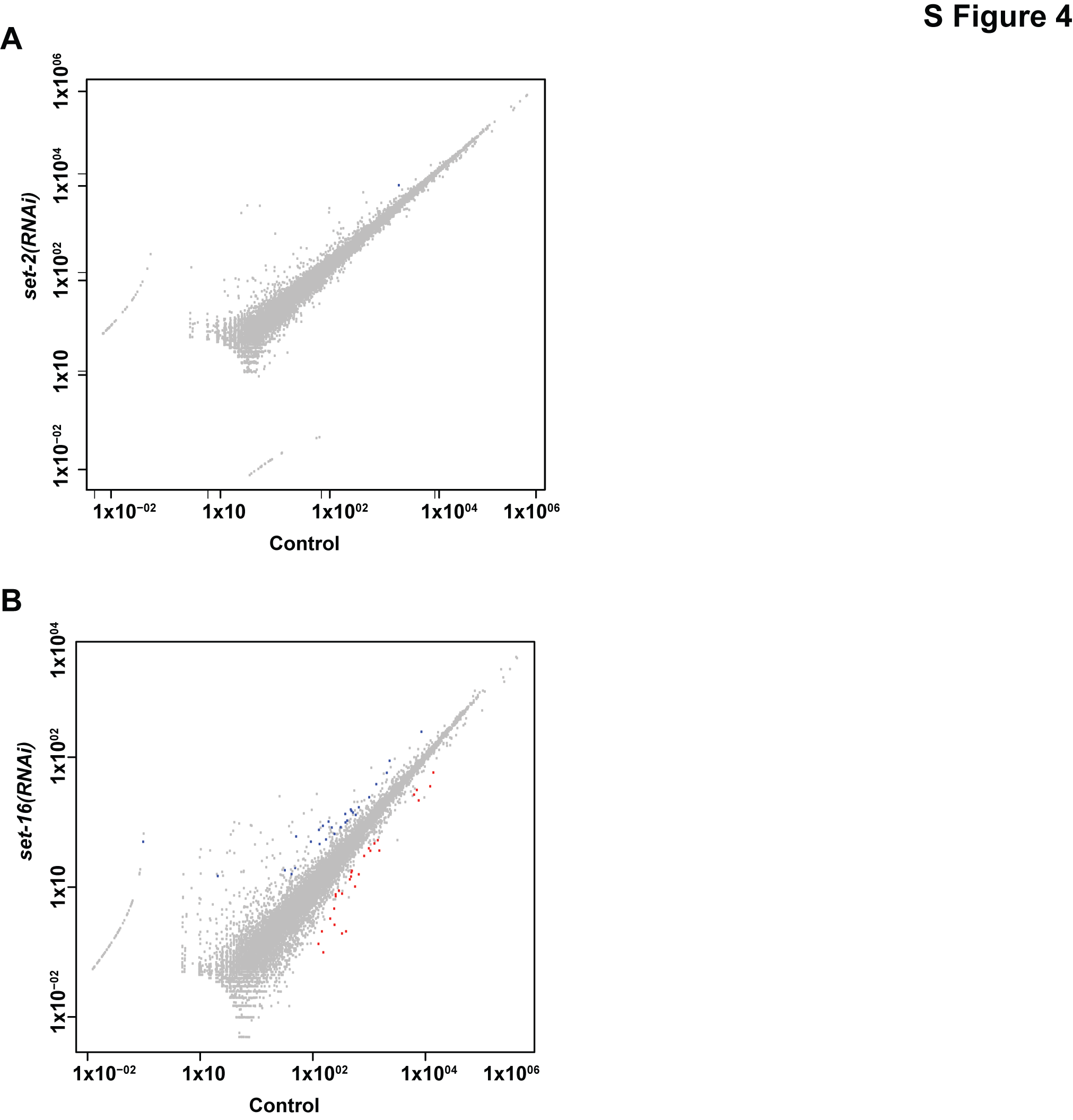

Supplement: S4 Fig — Scatter plot showing significantly up (red) or down (blue) regulated genes after set-2 (A) or set-16 (B) RNAi. (TIF) [file pgen.1007812.s004.tif]

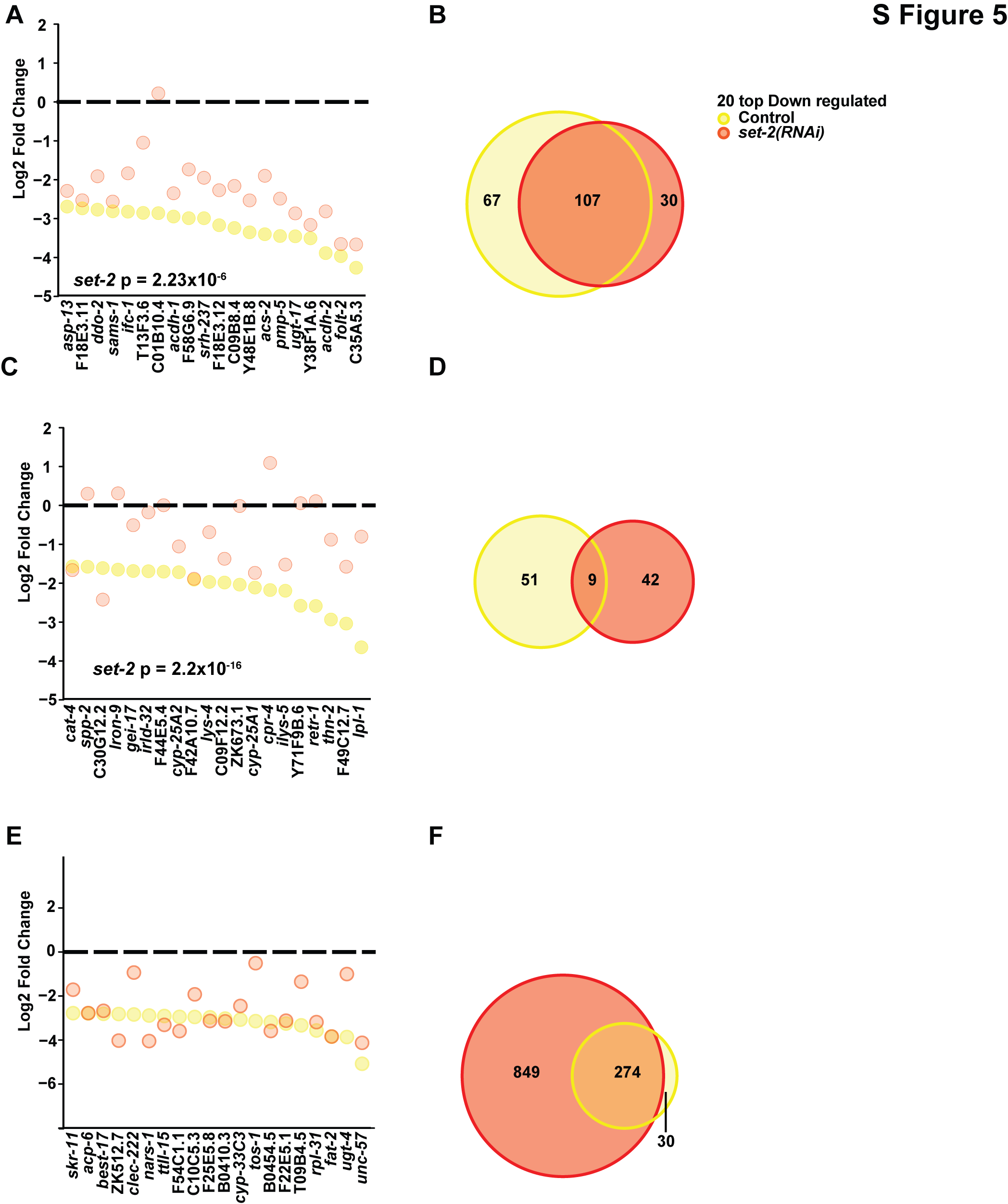

Supplement: S5 Fig — Strip plots of the lowest 20 expressed genes (A, C, E) and Venn diagrams (B, D, F) comparing control (yellow) and set-2(RNAi) (orange) animals after treatment with P. aeruginosa (A, B), R24 (C, D) or heat stress (E, F). RNA for sequencing was isolated from control, sams-1, set-2 and set-16 RNAi as a set for each stress. Therefore, control genes in A-F are the same as in S3 Fig for each stress. (TIF) [file pgen.1007812.s005.tif]

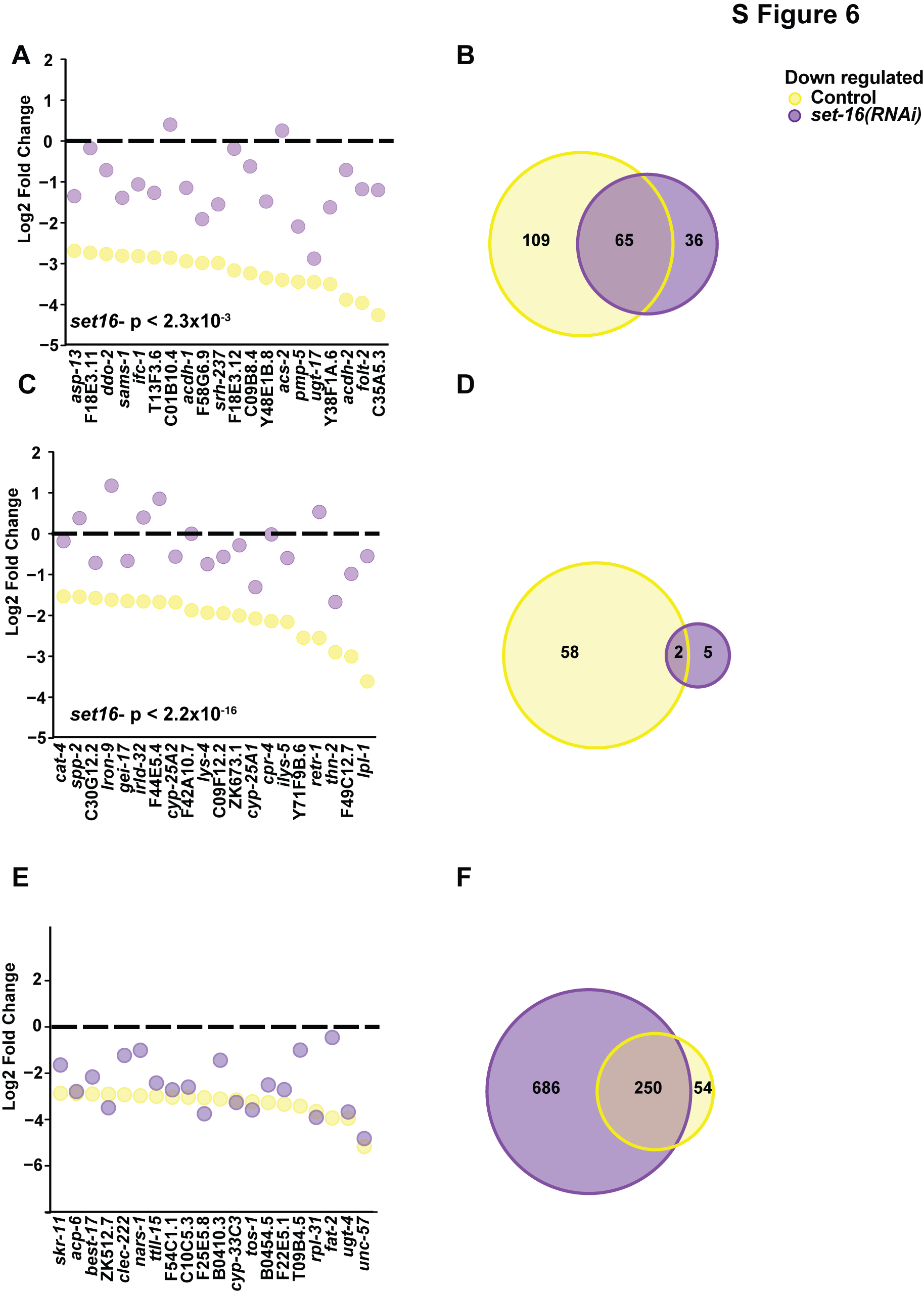

Supplement: S6 Fig — Strip plots of the lowest 20 expressed genes (A, C, E) and Venn diagrams (B, D, F) comparing control (yellow) and set-16(RNAi) (purple) animals after treatment with P. aeruginosa (A, B), R24 (C, D) or heat stress (E, F). RNA for sequencing was isolated from control, sams-1, set-2 and set-16 RNAi as a set for each stress. Therefore, control genes in A-F are the same as in S3 Fig for each stress. (TIF) [file pgen.1007812.s006.tif]

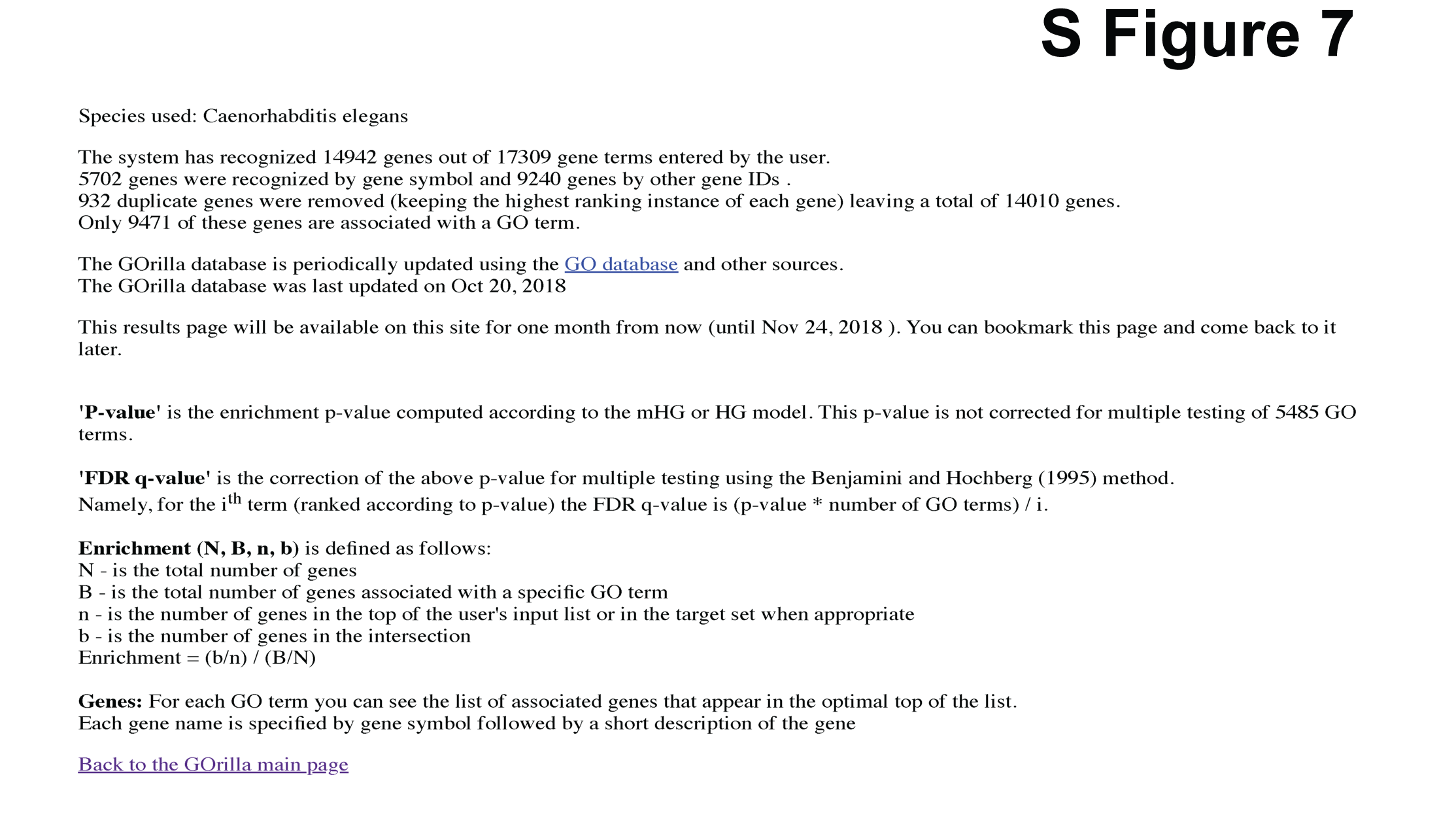

Supplement: S7 Fig — (TIF) [file pgen.1007812.s007.tif]

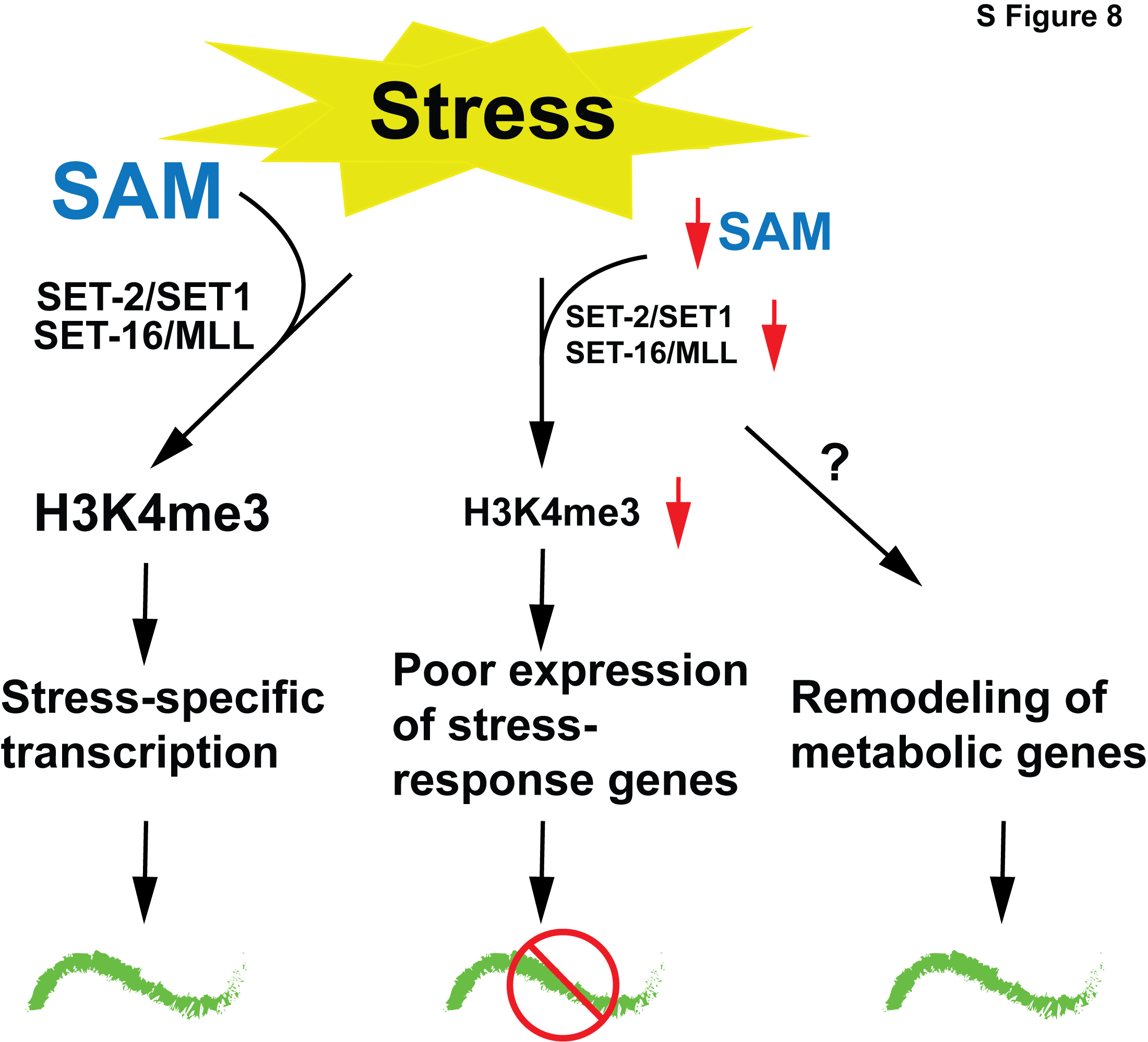

Supplement: S8 Fig — (TIF) [file pgen.1007812.s008.tif]
